# Frequency-dependent modulation of functional single cell oils in *Rhodotorula* sp. and *Aspergillus flavus* under alternating current stimulation

**DOI:** 10.1186/s13068-026-02757-3

**Published:** 2026-04-02

**Authors:** Hadeel El-Shall, Afaf A. Gliwan, Mamdouh M. Shawki, Marwa M. Eltarahony, Moataz M. Fahmy

**Affiliations:** 1 Environmental Biotechnology Department, Genetic Engineering and Biotechnology Research Institute, City of Scientific Research and Technological Applications (SRTA-City), Alexandria, Egypt; 2 Medical Biophysics Department, Medical Research Institute, Alexandria University, Alexandria, Egypt

**Keywords:** Alternating current stimulation, Single-cell oils, Lipids, Oleaginous microorganisms, Polyunsaturated fatty acids, Bioelectrochemistry, SDGs concepts*, Rhodotorula*, *Aspergillus*

## Abstract

Microbial lipid metabolism can be modulated by non-invasive physical stimuli, offering a sustainable route to tailor functional biomaterials. Here, we report for the first time the frequency-dependent modulation of single cell oils (SCOs) in the oleaginous yeast *Rhodotorula* sp. and the filamentous fungus *Aspergillus flavus* through alternating current (AC) electrostimulation during exponential and stationary growth phases. Cultures were exposed to AC frequencies ranging from 100 Hz to 1 MHz under nitrogen-limited conditions, and lipid yield, fatty acids (FAs) composition, and ultrastructural changes were assessed via gravimetric analysis, GC–MS, FTIR spectroscopy, and TEM imaging. Low-frequency stimulation (100 Hz–1 kHz) significantly enhanced lipid accumulation (up to 2.7-fold above controls) with enrichment in oleic acid, a monounsaturated fatty acid (MUFA). Mid-frequency exposure (10–100 kHz) favored MUFAs accumulation at early stages but later suppressed unsaturation, implying transient desaturase modulation. High-frequency treatment (1 MHz) selectively enriched polyunsaturated fatty acids (PUFAs), particularly linoleic acid (ω-6), during prolonged cultivation, linking oxidative stress responses to lipid remodeling. TEM revealed frequency-specific ultrastructural adaptations, including lipid droplet enlargement, membrane invaginations, and autophagic activity. Together, these results demonstrate that AC frequency acts as a tunable “metabolic switch” capable of enhancing both the quantity and quality of microbial lipids. This strategy offers strong potential for scalable production of biofunctional lipids relevant to biofuels, nutraceuticals, biomedical, and advanced biomaterial applications, which aligns with various United Nations Sustainable Development Goals (SDGs) including zero hunger (SDG 2), good health and well-being (SDG 3), and affordable and clean energy (SDG 7).

**Supplementary Information:**

The online version contains supplementary material available at 10.1186/s13068-026-02757-3.

## Introduction

In oleaginous microorganisms (such as bacteria, algae, filamentous fungi, and yeasts), lipogenesis is a frequently occurring phenomenon that is fueled by the acetyl-CoA system in the context of an imbalanced metabolism. The carbon supply is continuously used by the developing cells, which changes into neutral lipids that are then stored as an energy source. Lipogenic microorganisms store these neutral lipids intracellularly as lipid droplets known as microbial oils, also known as single cell oils (SCOs) [1–3]. SCOs may include sterols, polar lipids, hydrocarbons, pigments, triacylglycerols (TAGs), saturated fatty acids (SFAs), monounsaturated fatty acids (MUFAs), and polyunsaturated fatty acids (PUFAs) such as omega-3 (ω-3) and omega-6 (ω-6) linoleic acid. PUFAs play key roles in regulating membrane fluidity, stress adaptation, oxidative balance, and cellular signaling. Their biomedical importance, including roles in inflammation control, wound healing, and metabolic regulation, makes PUFAs-rich SCOs desirable for functional biomaterial applications [4–6].

The growing demand for sustainable energy sources has highlighted the importance of SCOs as alternative lipid feedstocks, as the FAs profile of these lipids makes them suitable not only for biofuel production but also for applications in nutraceuticals, cosmetics, detergents, and food industries [6, 7], representing a promising and eco-friendly solution due to their rapid production rates, scalability, and ability to accumulate high levels of lipids under controlled conditions [3, 4].

A wide range of microorganisms, including filamentous fungi, bacteria, yeasts, and microalgae, have been reported to produce intracellular lipids with diverse biochemical profiles efficiently. Among oleaginous yeasts, *Rhodotorula* species (family *Sporidiobolaceae*, Basidiomycota) are notable for their ability to accumulate lipids and produce valuable carotenoid pigments under stress conditions. These yeast cells are often red to orange and can tolerate a range of environmental stresses, contributing to their metabolic versatility [8, 9]. Similarly, the filamentous fungus *Aspergillus flavus* (family *Aspergillaceae*, Ascomycota) is a widespread environmental organism known for its high adaptability and bioconversion capacity, including potential for lipid accumulation under nitrogen-limited conditions [10, 11].

Lipid accumulation in oleaginous microorganisms typically occurs when nitrogen becomes limited in the presence of excess carbon, triggering redirection of carbon flux into FAs biosynthesis and storage lipid accumulation [12]. Efficient lipid recovery requires cell wall disruption, as these lipids are primarily stored intracellularly. Common mechanical or chemical methods include solvent extraction, ultrasonication, microwave-assisted lysis, and acid/base treatments. However, these often involve high energy input or generate hazardous waste [13, 14].

In recent years, electro-technological interventions have emerged as sustainable, non-invasive methods to influence microbial behavior, stimulate metabolite production, and enhance extraction processes. These include pulsed electric fields (PEF), direct current (DC), and alternating current (AC) applications [15, 16]. Compared to conventional methods, electric fields are considered low-cost, energy-efficient, and environmentally benign [17].

However, while most studies focus on the disruptive or extraction-enhancing effects of electric fields, the use of AC frequency tuning as a precise tool to control both yield and fatty acids profile in oleaginous species has not been reported. AC electric fields, in particular, may influence intracellular signaling, membrane potential, or enzymatic activity in frequency-dependent ways and potentially affecting lipid biosynthesis pathways [18, 19].

Therefore, the present study investigates for the first time the frequency-dependent modulation of SCOs production and lipid composition in *Rhodotorula* sp. and *A. flavus* under nitrogen-limited conditions. We applied AC stimulation across a range of low (100 Hz–1 kHz), mid (10–100 kHz), and high (1 MHz) frequencies during both exponential and stationary growth phases. *Rhodotorula* sp. (unicellular fungi or yeast) and *A. flavus* (multicellular filamentous fungus) were selected because both are well-established oleaginous microorganisms capable of accumulating TAGs- and PUFAs-rich SCOs. Together, they represent two distinct biological systems routinely maintained in our laboratory, enabling comparative evaluation of AC-induced lipid modulation across taxonomic groups. Lipid yield, fatty acids profiles, and ultrastructural features were analyzed using gravimetric methods, GC–MS, FTIR spectroscopy, and TEM imaging. Our findings reveal that AC frequency can be used as a tunable, low-energy tool to selectively enhance lipid yield or enrich specific fatty acid classes, with direct implications for biodiesel, nutraceutical, and functional biomaterial applications.

## Materials and methods

### Strain, culture media, and growth conditions

The yeast strain *Rhodotorula* sp. was isolated from coral reefs from Marsa Alam, Red Sea governorate, Egypt. This isolate has been characterized previously by its strong metabolic plasticity and classified as a high-performing oleaginous strain within local indoor culture collections. Its 18S rRNA gene sequence has been identified previously and deposited in the GenBank database under the accession number of MZ312359 [20]. Whereas, the fungal strain *A. flavus* was isolated from Mediterranean coastal sediments in Alexandria, Egypt. It has been examined previously and emphasized its outstanding bioremediating efficiency among existing indoor culture collections. Its 18S rRNA gene sequence has been identified and deposited in the GenBank database under the accession number of PP761263 [21].

For yeast propagation, 75 mL of yeast peptone dextrose (YPD) broth (1% yeast extract, 2% peptone, 2% glucose) was inoculated in 250 mL Erlenmeyer flasks and incubated for 48 h at 28 °C with shaking at 180 rpm. For *A. flavus*, 75 mL of Czapek-Dox broth, a defined medium suitable for fungal propagation under nitrogen-limited conditions (g/L: sucrose 30, sodium nitrate 2, dipotassium phosphate 1, magnesium sulfate 0.5, potassium chloride 0.5, ferrous sulfate 0.01), was used under similar incubation conditions. After incubation, each culture was centrifuged at 12,000 rpm for 10 min, washed twice with sterile distilled water, and resuspended in sterile water for subsequent use as a seed culture in lipid production experiments.

### AC exposure system

**Fig. 1:**
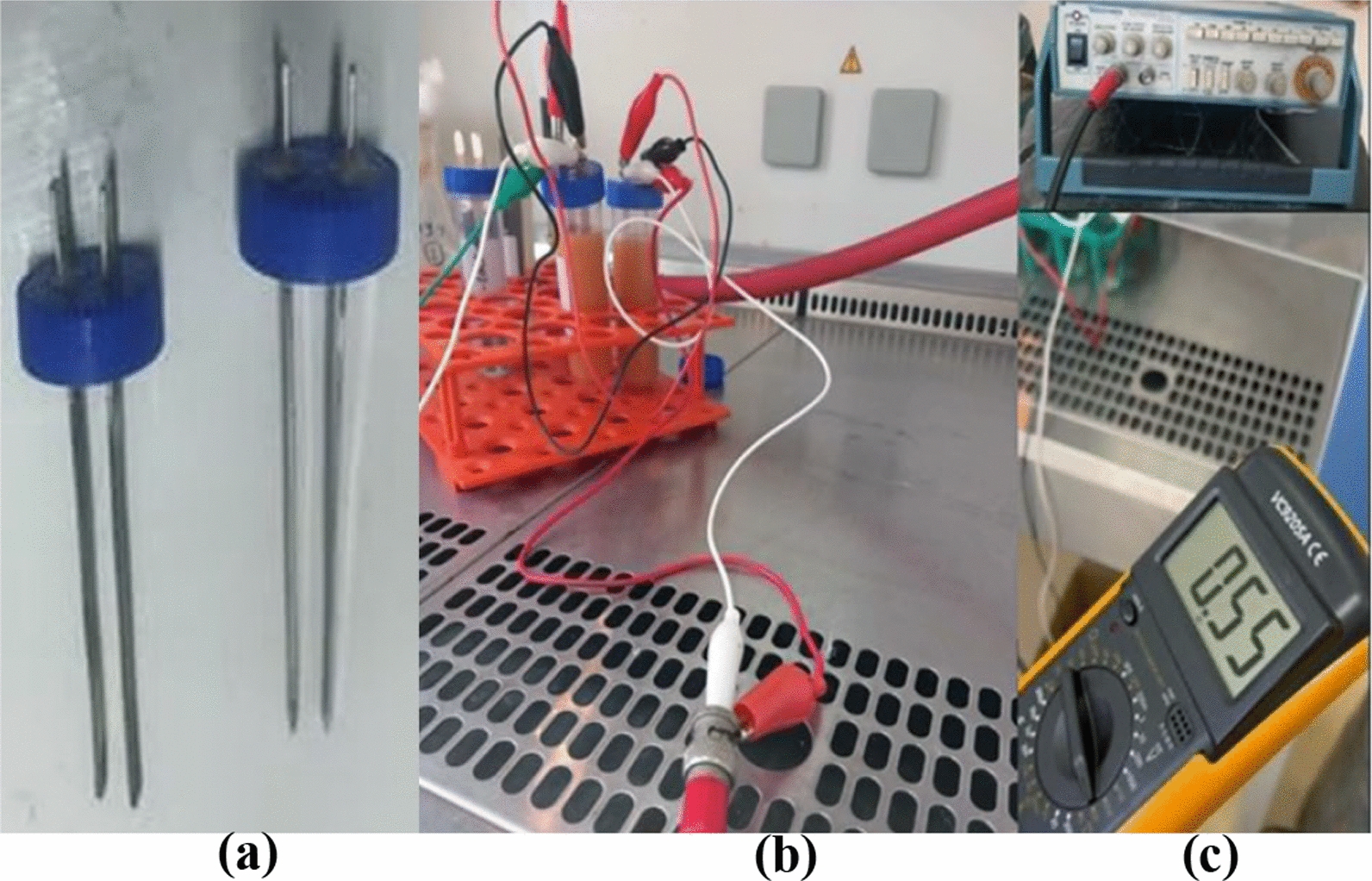
Photographic representation of the AC exposure setup, including **a** Electrodes before exposure, **b** Falcon tubes with cultures exposed, and **c** Function generator close-up, and voltmeter displaying 0.55 V

The present study aimed to evaluate the impact of different frequencies of alternating electric current (AC) on lipid accumulation and FAs profiles in two lipogenic microorganisms: the oleaginous yeast *Rhodotorula* sp. and the filamentous fungus *A. flavus*. The exposure protocol was performed for cultures at 18 h (18 h exposure) and 48 h of incubation (48 h exposure) to represent the exponential growth phase and the stationary growth phase, respectively [1, 22–24]. To implement this design, seed cultures of *Rhodotorula* sp. and *A. flavus* were separately prepared and inoculated into a nitrogen-limited modified YM medium, consisting of 0.5% yeast extract, 0.5% peptone, and 2% glucose. Cultures were incubated at 28 °C in an orbital shaker at 180 rpm. At the designated time points, each culture was subjected to a specific AC frequency field using a digital function generator (CALTEK, CA1640P-02, USA; serial number: 06mg0676), configured to deliver square pulses at about 0.5 V for 5 min. The selected low-voltage AC parameters were informed by preliminary optimization experiments in our laboratory, designed to avoid direct physical effects, including heating, electrolysis, or membrane perturbation, thereby ensuring that observed responses reflect metabolic modulation rather than electrical damage. Exposure was conducted via two solid Ag/AgCl rectangular electrodes (each 1 cm × 5.0 cm, with a total active area of 10 cm^2^) positioned 2 cm apart, inserted in parallel through holes in the lid of 50 mL falcon tubes and connected to the generator’s output terminals [25]. Control cultures (i.e., without any treatment) underwent an identical handling procedure as the treated groups, including transfer into the same type of conical tubes, contact with the same Ag/AgCl electrodes, and identical manipulation steps; however, the function generator remained switched OFF during this sham exposure. After the 5-min exposure, all cultures (i.e., treated and control) were immediately returned to incubation and allowed to continue growing until the end of the 120 h fermentation cycle, at which point biomass and lipid analyses were performed. The design of the exposure unit is shown in Fig. 1. Experimental groups (G1–G5) were exposed to one of five AC frequencies (100 Hz, 1 kHz, 10 kHz, 100 kHz, or 1 MHz), while the unexposed control group (G0) was maintained under identical conditions without electrical exposure.

### Determination of biomass concentration, lipid concentration, and lipid content

The biomass was determined at the end of incubation time (120 h). The cultures were harvested in triplicate by centrifugation at 12,000 rpm for 10 min. The resulting biomass pellets were washed three times with distilled water to remove residual medium components and then dried at 60 °C until a constant weight. Biomass dry weight (DW) was determined gravimetrically by subtracting the weight of the empty container from the weight of the container plus dried biomass. Biomass concentration was expressed as grams of DW per liter of culture (g DW/L) [26].

 Lipids were extracted and quantified from dried biomass using a chloroform–methanol mixture (2:1, v/v) as described by Folch et al. [27]. Briefly, the dried biomass was ground and mixed with the solvent, agitated at 200 rpm for 20 min at room temperature, and centrifuged at 6000 rpm for 10 min. The extraction was repeated twice to maximize recovery. The pooled organic phases were evaporated to dryness under reduced pressure, and the lipid residue was weighed. Lipid concentration was calculated as grams of lipid per liter of culture (g/L).

Lipid content was calculated from biomass and lipid concentrations using the following equation:

1 $$\text{Lipid content }({\% DW}) = \frac{Lipid \;concentration \;g/L}{Biomass\; concentration \;g DW/L}\times 100$$

### Characterization of the extracted SCOs and microbial ultrastructure

The extracted lipids were then subjected to acid-catalyzed transesterification by reacting with 20 mL of methanol and 2 mL of concentrated sulfuric acid at 70 °C for 2 h. After cooling to room temperature, the reaction mixture was transferred to a separating funnel for phase separation, and the upper FA methyl ester layer was collected and stored [28]. The extracted SCOs from all treatments were characterized by gas chromatography–mass spectrometry (GC–MS) and fourier transform Infrared spectroscopy (FTIR). Structural changes in the microbial cells were examined using transmission electron microscopy (TEM).

#### Gas chromatography–mass spectrometry (GC–MS)

To identify and quantify the saturated and unsaturated FAs, the transesterified lipid samples were analyzed using an Agilent 6890 gas chromatograph equipped with a 15 m Alltech EC-5 capillary column (250 µm I.D., 0.25 µm film thickness) and a straight deactivated 2 mm direct injector liner. The injection temperature was set to 250 °C, the detector temperature to 280 °C, and the oven was initially held at 35 °C for 2 min before ramping to 300 °C for 23 min. Helium was used as the carrier gas at a constant flow rate of 1 mL/min, with a split ratio of 10:1. The injection volume was 2 µL. FAs were identified by comparing chromatographic retention times (RT) and mass spectra with entries from the WILEY 09 and NIST 11 mass spectral libraries. Quantification was based on individual peak areas, and results were expressed as the relative area percentage (area %) of each FA methyl ester [29, 30].

#### Fourier transform infrared spectroscopy (FTIR)

Extracted lipid samples were analyzed by FTIR using a Shimadzu FTIR-8400S spectrometer. Samples were mixed with KBr and pressed into discs. FTIR spectra were recorded from 4000 to 400 cm⁻^1^ at a resolution of 4 cm⁻^1^ to identify characteristic lipid functional groups [31].

#### Transmission electron microscopy (TEM)

For ultrastructural analysis, biomass samples of both *Rhodotorula* sp*.* and *A. flavus* at each AC frequency were fixed in Karnovsky’s solution and post-fixed in 1% osmium tetroxide. Samples were dehydrated through an ethanol-acetone series, embedded in EPON resin, and ultrathin sections (~ 60 nm) were cut using a Leica Ultracut UCT ultramicrotome. Sections were stained with lead citrate and uranyl acetate and imaged using a JEOL JEM-1230 electron microscope at 200 kV [32]. For each condition, approximately 15–20 cells were examined across multiple grid fields. Representative images were selected based on common consistent morphological features among the majority of examined cells in the investigated fields.

### Statistical analysis of the data

All quantitative data (biomass yield, lipid content, and lipid yield) were derived from three independent biological replicates and expressed as mean ± standard deviation (SD). Statistical comparisons between groups were performed using one-way ANOVA in GraphPad InStat. A p-value ≤ 0.05 was considered statistically significant. GC–MS, FTIR, and TEM data were used for qualitative and comparative interpretation and were not subjected to statistical analysis.

## Results and discussion

### Biomass growth and lipid accumulation under AC frequency stimulation

#### Biomass growth and lipid accumulation of *Rhodotorula* sp. for 18 h exposure

Biomass concentration, lipid concentration, and lipid content of *Rhodotorula* sp. for 18 h exposure are presented in Table 1. The control culture reached 7.0 ± 0.33 g DW/L, whereas electrically stimulated groups ranged from 6.08 ± 0.13 g DW/L (100 Hz) to 4.80 ± 0.10 g DW/L (1 MHz), representing reductions of 13–31%. Such decreases may reflect physiological stress induced by oscillating electric fields [33], which can increase maintenance energy demands or impair cell division rates in electrostressed microorganisms [34].

**Table 1:** Biomass concentration, lipid concentration, and lipid content in *Rhodotorula* sp. Cells for 18 h exposure

	Control	100 Hz	1 kHz	10 kHz	100 kHz	1 MHz
Biomass Concentration (g Dw/L)	7.00 ± 0.33	6.08 ± 0.13	5.88 ± 0.15	5.82 ± 0.15	5.86 ± 0.14	4.80 ± 0.10
Lipid Concentration (g/L)	0.50 ± 0.06	1.04 ± 0.14	1.00 ± 0.12	1.00 ± 0.15	1.00 ± 0.18	0.70 ± 0.09
Lipid Content (% Dw)	7.14 ± 0.45	17.11 ± 1.91	17.00 ± 1.82	17.18 ± 1.94	17.06 ± 2.00	14.58 ± 1.61

In contrast, lipid production showed clear stimulation under AC exposure. The control exhibited 0.50 ± 0.06 g/L (7.14 ± 0.45% DW), while all frequencies except 1 MHz more than doubled lipid yield, reaching ~ 1.00–1.04 g/L (~ 17% DW). This increase was statistically significant (p < 0.05) compared to the control. Even at 1 MHz, despite a 31% biomass reduction, lipid content remained elevated (14.58 ± 1.61% DW), indicating enhanced biosynthetic efficiency per unit biomass.

The inverse relationship between biomass and lipid content suggests a metabolic reprogramming from growth to storage lipid accumulation. This behavior is consistent with stress-induced lipogenesis, where cells redirect carbon flux toward TAGs synthesis under environmental stressors [35]. The strongest responses occurred at low to mid frequencies (100 Hz–100 kHz), possibly reflecting frequency-dependent modulation of membrane potential or activation of lipid biosynthetic pathways [36].

#### Biomass growth and lipid accumulation of *Rhodotorula* sp. for 48 h exposure

Biomass concentration in the control culture reached 9.40 ± 0.57 g DW/L, higher than at 18 h exposure (Table 2). AC stimulation again caused frequency-dependent biomass reductions, with the largest declines at 10 kHz (6.44 ± 0.36 g DW/L; − 31%) and 100 kHz (6.04 ± 0.31 g DW/L; − 36%) compared to control (p < 0.05). These declines align with reports that electric fields can disrupt cell-cycle progression or increase maintenance energy demands in microorganisms, leading to lower biomass yields [37, 38].

**Table 2:** Biomass concentration, lipid concentration, and lipid content in *Rhodotorula* sp. cells for 48 h exposure

	Control	100 Hz	1 kHz	10 kHz	100 kHz	1 MHz
Biomass concentration (g Dw/L)	9.40 ± 0.57	8.40 ± 0.61	7.20 ± 0.43	6.44 ± 0.36	6.04 ± 0.31	6.20 ± 0.42
Lipid concentration (g/L)	0.96 ± 0.11	1.62 ± 0.20	1.32 ± 0.18	1.12 ± 0.15	0.92 ± 0.12	0.84 ± 0.07
Lipid content (% Dw)	10.21 ± 0.55	19.29 ± 3.80	18.33 ± 3.40	17.39 ± 3.10	15.23 ± 1.37	13.55 ± 1.22

For lipid production, the control reached 0.96 ± 0.11 g/L (10.21 ± 0.55% DW). Most AC treatments, particularly 100 Hz (1.62 ± 0.20 g/L; 19.29 ± 3.80% DW) and 1 kHz (18.33 ± 3.40% DW), significantly (p < 0.05) enhanced lipid yield and content. Higher frequencies (100 kHz, 1 MHz) produced smaller increases, suggesting diminished lipogenic stimulation at very rapid oscillation rates.

The trend of reduced biomass yet elevated lipid proportion mirrors the 18 h exposure results, indicating that AC exposure consistently redirects metabolic flux from proliferation toward lipid storage across growth phases [39, 40]. Notably, while absolute biomass increased between 18 h exposure and 48 h exposure in both control and treated cultures, the relative lipid enhancement remained most pronounced at low frequencies. This consistency suggests that early metabolic reprogramming persists into the stationary phase, with low-frequency AC delivering the best balance between biomass retention and lipid enrichment. While biomass remained relatively stable at lower frequencies across both time points, lipid accumulation was consistently higher at 48 h exposure, particularly at 100 Hz (2.64 g/L vs. 2.12 g/L at 18 h exposure). Lipid content also increased more sharply at 48 h exposure, suggesting that extended electrical stimulation strengthens the metabolic shift toward storage lipid without severely compromising growth at optimal frequencies.

#### Biomass growth and lipid accumulation of *A. flavus* for 18 h exposure

Electrical stimulation of *A. flavus* at 18 h exposure significantly affected both biomass yield and lipid metabolism (Table 3). Biomass concentration decreased progressively with increasing AC frequency, from 6.92 ± 0.39 g DW/L in the control to 5.06 ± 0.14 g DW/L at 1 MHz (p < 0.05 for all treatments vs. control). This reduction is consistent with reports that electric fields can impose cellular stress and disrupt growth kinetics through electroporation and membrane perturbation [41, 42].

**Table 3:** Biomass concentration, lipid concentration, and lipid content in *A. flavus* for 18 h exposure

	Control	100 Hz	1 kHz	10 kHz	100 kHz	1 MHz
Biomass concentration (g DW/L)	6.92 ± 0.39	6.22 ± 0.28	5.4 ± 0.16	5.26 ± 0.14	5.3 ± 0.12	5.06 ± 0.14
Lipid concentration (g/L)	0.58 ± 0.07	1.1 ± 0.12	0.92 ± 0.08	0.88 ± 0.09	0.8 ± 0.08	0.8 ± 0.1
Lipid content (% DW)	8.38 ± 2.05	17.68 ± 1.14	17.03 ± 1.99	16.73 ± 2.16	15.09 ± 1.17	15.81 ± 2.42

Despite the lower biomass, lipid concentration increased markedly at 100 Hz (1.10 ± 0.12 g/L) compared with the control (0.58 ± 0.07 g/L), representing ~ 90% enhancement (p < 0.01). Even at higher frequencies, lipid levels remained at or above control values, indicating that electric stimulation triggered a metabolic shift toward lipid biosynthesis rather than cell proliferation [43].

Lipid content (% DW) rose from 8.38 ± 2.05% (control) to 17.68 ± 1.14% at 100 Hz (p < 0.01) and remained elevated (> 15%) across frequencies. The frequency-specific effects observed here are consistent with established models of stress-induced lipogenesis, where changes in membrane potential and ion flux act as primary triggers for lipid biosynthetic enzymes. In oleaginous yeasts, activation of ATP-citrate lyase and malic enzyme under nitrogen limitation drives increased cytosolic acetyl-CoA and NADPH availability, favoring triacylglycerol accumulation. Low-frequency AC stimulation may further enhance this flux by modulating membrane-associated redox systems, while high-frequency stimulation could influence desaturase activity, thereby shifting fatty acids profiles toward MUFAs or PUFAs enrichment [44]. The dual outcome of lower biomass but higher lipid fraction parallels findings in other oleaginous fungi exposed to physical stimuli, where lipid production becomes decoupled from growth as an adaptive energy-storage response [44].

#### Biomass growth and lipid accumulation of *A. flavus* for 48 h exposure

*A. flavus* exhibited for 48 h exposure, a frequency-dependent shift in biomass, lipid concentration, and lipid content (Table 4). Control biomass was 8.10 ± 0.59 g DW/L, while exposure to higher frequencies (≥ 10 kHz) led to significant reductions, with the lowest biomass measured at 1 MHz (4.48 ± 0.27 g DW/L; p < 0.05). This pattern follows the effect seen at 18 h exposure and aligns with reports that electric fields impair fungal growth through altered membrane potential, oxidative stress, and nutrient-transport disruption [45, 46].

**Table 4:** Biomass concentration, lipid concentration, and lipid content in *A. flavus* for 48 h exposure

	Control	100 Hz	1 kHz	10 kHz	100 kHz	1 MHz
Biomass Concentration (g DW/L)	8.1 ± 0.59	7.74 ± 0.46	7 ± 0.50	5.42 ± 0.38	5.3 ± 0.22	4.48 ± 0.27
Lipid Concentration (g/L)	0.76 ± 0.14	1.6 ± 0.14	1.4 ± 0.15	1.06 ± 0.06	0.88 ± 0.08	0.68 ± 0.05
Lipid Content (% DW)	9.38 ± 1.05	20.67 ± 0.58	20 ± 0.72	19.56 ± 0.27	16.60 ± 0.82	15.18 ± 0.2

Lipid accumulation was markedly enhanced at low and intermediate frequencies. The highest lipid concentration (1.60 ± 0.14 g/L) occurred at 100 Hz, about a 110% increase over the control (*p* < 0.01). Lipid content peaked at 100 Hz (20.67 ± 0.58% DW), more than doubling the control (9.38 ± 1.05% DW). Elevated lipid fractions were retained even at ≥ 100 kHz despite substantial biomass loss, indicating that surviving cells under electrical stress preserve high lipid content. These observations may reflect shifts in cellular redox conditions and triacylglycerol (TAG) synthesis activity, as suggested in prior studies on electrically modulated lipid metabolism; however, direct mechanistic measurements were beyond the scope of the present work [45].

From 18 h exposure to 48 h exposure, *A. flavus* showed an overall increase in biomass in both control and treated cultures, but biomass suppression by high-frequency stimulation became more pronounced at the later time point. Lipid concentration and content were consistently higher at 48 h exposure, especially at 100 Hz, suggesting that the stress-induced metabolic redirection toward lipid synthesis persisted and intensified with longer cultivation.

#### Comparison between biomass growth and lipid accumulation of *Rhodotorula* sp. and *A. flavus*

Both species showed a similar overall response to AC electrical stimulation: low-frequency exposure, especially at 100 Hz, increased lipid accumulation, while higher frequencies decreased biomass. However, *Rhodotorula* sp. maintained higher biomass at both time points, indicating greater tolerance to electrical stress. In contrast, *A. flavus* experienced stronger biomass suppression at ≥ 10 kHz but showed a larger proportional increase in lipid content, particularly at 48 h exposure. Over time, lipid production increased in both organisms, but *Rhodotorula* sp. maintained a better balance between growth and lipid accumulation, while *A. flavus* prioritized lipid storage more strongly as a stress response. These differences emphasize the importance of species-specific optimization of electrical stimulation parameters to maximize SCOs productivity.

### Fatty acids profile changes gas chromatography–mass spectrometry (GC–MS)

#### Fatty acids profile of *Rhodotorula* sp. at 18 h exposure

The GC–MS analysis of *Rhodotorula* sp. lipids for 18 h exposure in their response to electrical stimulation revealed notable variations in the relative abundance of key FA species across different AC frequencies (Table 5). Full GC–MS chromatograms of *Rhodotorula* sp. at 18 h exposure are provided in Supplementary Figure S1. Full GC–MS data, including RT and molecular formula, are provided in Supplementary Table S1.

**Table 5:** Major Fatty Acids Identified in *Rhodotorula* sp. Cells for 18 h exposure

Compound name	Common name	Type of FA	Control group	100 Hz	1 kHz	10 kHz	100 kHz	1 MHz
Hexadecanoic acid	Palmitic acid	SFA	11.54	15.28	14.85	12.20	13.59	14.16
Hexadecanoic acid, methyl ester	Methyl palmitate	SFA	–	–	–	0.81	0.93	–
9-Octadecenoic acid	Oleic acid (omega-9)	MUFA	23.65	22.47	26.33	28.62	27.14	20.26
9-Octadecenoic acid, methyl ester	Methyl oleate	MUFA	–	–	–	1.29	–	2.42
9,12-Octadecadienoic acid	Linoleic acid (omega-6)	PUFA	0.18	0.24	0.35	0.25	0.26	11.26
9,12-Octadecadienoic acid, methyl ester	Methyl linoleate	PUFA	–	–	–	0.94	–	–
Octadecanoic acid	Stearic acid	SFA	6.70	5.15	4.94	2.19	3.08	2.76
Octadecanoic acid, methyl ester	Methyl stearate	SFA	–	–	–	0.78	1.21	2.38
9,15-Octadecadienoic acid	–	PUFA	–	–	–	–	–	0.52
9-Hexadecenoic acid	Palmitoleic acid	MUFA	1.24	0.82	0.66	0.33	0.42	0.79
Tetradecanoic acid	Myristic acid	SFA	0.54	0.86	0.71	0.75	0.82	0.63
Octadecanoic acid, 9,10-dihydroxy-, methyl ester	Methyl 9,10- dihydroxystearate	SFA	–	–	–	–	–	0.82

Values represent Area % obtained from GC–MS analysis. Common names and functional classifications (SFA, MUFA, PUFA) are provided

A total of 11 major FA compounds were detected, with saturation types classified into saturated FAs (SFAs), monounsaturated FAs (MUFAs), and polyunsaturated FAs (PUFAs). These are further summarized in Table 6 below.

**Table 6:** Fatty Acid Class Distribution in *Rhodotorula* sp. at 18 h Exposure (Area %)

Frequency	Total SFAs (%)	Total MUFAs (%)	Total PUFAs (%)	Total UFAs (%)
Control	18.78	24.89	0.18	25.07
100 Hz	22.11	23.29	0.24	23.53
1 kHz	21.24	27.00	0.35	27.35
10 kHz	17.92	30.24	1.19	31.43
100 kHz	20.63	27.56	0.26	27.82
1 MHz	20.75	23.47	11.78	35.25

Values represent the summed relative abundances of fatty acid classes

UFAs = MUFAs + PUFAs

At 18 h exposure, *Rhodotorula* sp. lipids were mainly composed of MUFAs, primarily oleic acid (ω-9), which reached 30.24% under 10 kHz stimulation—representing a 21% increase over the control (23.65%). Oleic acid is valued in biodiesel for its oxidative stability and cold-flow properties and is also significant for its anti-inflammatory and cardioprotective roles [7, 16]. Our findings suggest that mid-frequency AC stimulation enhances MUFAs biosynthesis, possibly by modulating membrane enzyme activity or lipid flux [33, 34]. In contrast, PUFAs levels remained low except at 1 MHz, where linoleic acid (ω-6) surged dramatically to 11.26%, a 62-fold increase compared to the control. These results highlight potential biomedical uses, considering omega-6's roles in immune modulation and skin health [34]. SFAs levels stayed stable across treatments, indicating that AC mainly influences FAs unsaturation patterns rather than overall diversity.

#### Fatty acids profile of *Rhodotorula* sp. for 48 h exposure

For 48 h exposure, further shifts in FAs composition were observed (Tables 7 and 8). Supplementary Figure S2 and Table S2 provide full chromatographic and molecular data.

**Table 7:** Major fatty acids identified in *Rhodotorula* sp. cells for 48 h exposure

Compound name	Common name	Type of FA	Control group	100 Hz	1 kHz	10 kHz	100 kHz	1 MHz
Hexadecanoic acid	Palmitic acid	SFA	11.9	13.38	13.05	13.78	6.44	19.45
9-Octadecenoic acid	Oleic acid (omega-9)	MUFA	24.2	13.72	11.23	9.21	6.08	12.57
9-Octadecenoic acid, methyl ester	Methyl oleate	MUFA	–	0.50	–	0.82	–	1.39
9,12-Octadecadienoic acid	Linoleic acid (omega-6)	PUFA	0.18	7.58	6.24	4.83	3.03	19.18
Octadecanoic acid	Stearic acid	SFA	6.7	2.7	3.3	2.68	1.09	3.28
Tetradecanoic acid	Myristic acid	SFA	0.54	0.72	0.45	–	–	–
Octadecanoic acid, 9,10-dihydroxy-, methyl ester	Methyl 9,10-dihydroxystearate	SFA	0.82	0.66	–	0.37	–	–

**Table 8:** Fatty acid class distribution in *Rhodotorula* sp. for 48 h exposure (area %)

Frequency	Total SFAs (%)	Total MUFAs (%)	Total PUFAs (%)	Total UFAs (%)
Control	19.96	24.20	0.18	24.38
100 Hz	17.46	14.22	7.58	21.80
1 kHz	16.80	11.23	6.24	17.47
10 kHz	16.83	10.03	4.83	14.86
100 kHz	7.53	6.08	3.03	9.11
1 MHz	22.73	13.96	19.18	33.14

At 48 h exposure, MUFAs content decreased across all frequencies compared to 18 h exposure, with the lowest level at 100 kHz (6.08%). Meanwhile, PUFAs (particularly linoleic acid) showed a pronounced increase at 1 MHz (19.18%), suggesting a delayed or stress-induced shift favoring PUFAs biosynthesis during stationary growth phase. SFAs levels remained largely stable, though a marked increase at 1 MHz (22.73%) likely compensates for MUFAs depletion. Low total unsaturated fatty acids (UFAs) at mid-range frequencies (e.g., 9.11% at 100 kHz) were also noticed. The pronounced reduction in total UFAs at 100 kHz may indicate frequency-selective interference with desaturase-mediated lipid modification. Since Δ9-desaturase and related enzymes are membrane-bound and depend on tightly regulated electron transfer processes, oscillatory electric fields within this frequency range may perturb membrane redox coupling efficiency or transmembrane potential gradients required for double-bond insertion. Such selective suppression of desaturation would explain the sharp decline in UFAs without a proportional decrease in total lipid accumulation. Identification of such inhibitory frequency windows may be particularly relevant for frequency-guided metabolic tuning in future bioelectrochemical process optimization [34, 36].

#### Fatty acids profile of *A. flavus* at 18 h exposure

Our GC–MS analysis of *A. flavus* lipids after 18 h exposure to electrical stimulation demonstrated significant shifts in saturated and UFA species across frequency treatments, suggesting modifications in metabolic and biosynthetic regulation (Tables 9 and 10). Supplementary Figure S3 and Table S3 present chromatograms and detailed data, respectively.

**Table 9:** Major Fatty Acid Compounds Detected in *A. flavus* at 18 h exposure (Area %)

Compound name	Common name	Type of FA	Control	100 Hz	1 kHz	10 kHz	100 kHz	1 MHz
Hexadecanoic acid, methyl ester	Methyl palmitate	SFA	14.90	–	20.56	24.22	6.44	15.20
9-Octadecenoic acid, methyl ester	Methyl oleate	MUFA	7.02	8.68	–	–	4.14	7.48
11-Octadecenoic acid, methyl ester	–	MUFA	–	–	10.93	10.97	–	–
9,12-Octadecadienoic acid, methyl ester	Methyl linoleate	PUFA	7.15	1.74	3.16	1.75	3.03	3.17
11,14-Eicosadienoic acid, methyl ester	–	PUFA	–	0.36	0.41	–	–	0.30
Octadecanoic acid, methyl ester	Methyl stearate	SFA	9.33	11.50	15.48	15.86	1.09	11.49
Tetradecanoic acid, methyl ester	Methyl myristate	SFA	–	1.52	0.97	0.88	–	1.27
Pentadecanoic acid, methyl ester	–	SFA	–	1.00	0.83	0.87	–	0.88
Heptadecanoic acid, methyl ester	–	SFA	–	0.78	–	0.58	–	1.04
Eicosanoic acid, methyl ester	–	SFA	–	0.67	0.70	–	–	–
Nonadecanoic acid, methyl ester	–	SFA	–	–	–	–	–	0.29
Docosanoic acid, methyl ester	–	SFA	0.23	0.26	–	–	–	–
Tetracosanoic acid, methyl ester	–	SFA	–	0.62	–	0.58	–	0.54

**Table 10:** Summary of fatty acid categories (% Area) in *A. flavus* at 18 h exposure

Frequency	Total SFAs (%)	Total MUFAs (%)	Total PUFAs (%)	Total UFAs (%)
Control	24.46	7.02	7.15	14.17
100 Hz	17.35	8.68	2.10	10.78
1 kHz	38.57	10.93	3.57	14.50
10 kHz	42.11	10.97	1.75	12.72
100 kHz	7.53	4.14	3.03	7.17
1 MHz	30.71	7.48	3.47	10.95

At 18 h exposure, SFAs were dominant in nearly all groups, reaching peaks of 42.11% at 10 kHz and 38.57% at 1 kHz, significantly exceeding the control (24.46%). These increases were mainly due to higher levels of methyl palmitate and methyl stearate, which enhance biodiesel's thermal and oxidative stability [36]. MUFAs, primarily methyl oleate and 11-octadecenoic acid methyl ester (ω-9), increased at mid-range frequencies (1–10 kHz), indicating stimulation of Δ9-desaturase activity or MUFAs biosynthesis pathways [38, 39]. PUFAs levels, mainly methyl linoleate (ω-6), were highest in the control group (7.15%) but generally decreased with stimulation, possibly because of PUFAs oxidation or degradation caused by electrical stress [40].

#### Fatty acids profile of *A. flavus* for 48 h exposure

At 48 h exposure, further alterations in FAs composition were observed (Tables 11 and 12). Supplementary Figure S4 and Table S4 present chromatograms and detailed data, respectively.

**Table 11:** Major fatty acid compounds detected in *A. flavus* for 48 h exposure

Compound name	Common name	Type	Control	100 Hz	1 kHz	10 kHz	100 kHz	1 MHz
Hexadecanoic acid, methyl ester	Methyl palmitate	SFA	18.00	21.68	14.76	12.66	21.56	25.96
Octadecanoic acid, methyl ester	Methyl stearate	SFA	10.50	15.21	11.84	7.78	18.09	18.45
Tetradecanoic acid, methyl ester	Methyl myristate	SFA	1.00	1.45	1.66	2.53	1.04	1.28
Methyl pentadecanoate	–	SFA	1.00	1.14	0.89	0.53	0.94	0.76
Methyl heptadecanoate	–	SFA	0.80	1.16	1.00	1.11	1.11	0.75
Methyl tetracosanoate	–	SFA	0.50	1.14	0.62	0.61	0.64	0.49
Methyl eicosanoate	–	SFA	0.60	0.91	1.00	1.13	1.13	–
9-Octadecenoic acid, methyl ester	Methyl oleate	MUFA	8.00	10.23	5.45	3.61	11.14	2.72
11-Octadecenoic acid, methyl ester	–	MUFA	6.00	15.21	5.45	3.61	11.14	0.77
10-Octadecenoic acid, methyl ester	–	MUFA	0.50	0.85	–	0.77	–	0.77
(9,12-Octadecadienoic acid)	Methyl linoleate	PUFA	1.00	1.90	2.18	3.22	4.16	1.95
7,10-Octadecadienoic acid, methyl ester	–	PUFA	0.50	0.85	0.34	0.86	0.86	0.55

**Table 12:** Summary of Fatty Acid Categories (% Area) in *A. flavus* at 48 h exposure

Frequency	Total SFAs (%)	Total MUFAs (%)	Total PUFAs (%)	Total UFAs (%)
Control	32.40	14.50	1.50	16.00
100 Hz	42.69	26.29	2.75	29.04
1 kHz	31.77	10.90	2.52	13.42
10 kHz	26.95	7.99	4.08	12.07
100 kHz	44.55	22.28	5.02	27.30
1 MHz	47.24	4.26	2.50	6.76

At 48 h exposure, *A. flavus* showed a high proportion of SFAs, mainly methyl palmitate and methyl stearate, which together made up over 30% of the lipid profile in all groups. SFAs levels were highest at 1 MHz (47.24%), indicating stress-related lipid rigidification during stationary growth phase. In contrast, MUFAs, especially methyl oleate and 11-octadecenoic acid, peaked at 100 Hz (26.29%), suggesting that low-frequency stimulation might help maintain Δ9-desaturase activity or support pathways for unsaturated lipid biosynthesis.

PUFAs, including methyl linoleate (ω-6), increased most notably at 100–100 kHz (up to 5.02%). This response may indicate a late-onset oxidative stress adaptation involving redox-regulated FAs desaturation. However, the overall UFAs content sharply declined at 1 MHz (6.76%), suggesting possible enzyme inhibition or oxidative inactivation under high-frequency fields.

#### Comparison between fatty acids profile changes in *Rhodotorula* sp. and *A. flavus*

The GC–MS analysis of *Rhodotorula* sp. and *A. flavus* revealed frequency and time-dependent remodeling of FAs profiles under AC electrical stimulation. At 18 h exposure, both organisms exhibited increased saturated and MUFAs levels at mid-range frequencies (1–10 kHz), with *Rhodotorula* sp. notably enhancing oleic acid (MUFAs) content, beneficial for biodiesel quality and biomedical relevance. PUFAs generally remained low at early exposure times but showed dramatic increases at high frequency (1 MHz) at 48 h exposure, indicating a late-stage metabolic shift possibly linked to oxidative stress adaptation and membrane remodeling.

In *Rhodotorula* sp*.*, MUFAs content peaked early (18 h exposure, 10 kHz), then declined with time as PUFAs synthesis increased, especially linoleic acid at 1 MHz. The observed biphasic response is consistent with patterns reported in oleaginous microorganisms, where electrical or environmental cues modulate desaturase activity and lipid metabolic flux; however, direct assessment of desaturase regulation was not performed in this study. *A. flavus* followed a similar trend, with early enrichment of SFAs at 1–10 kHz and enhanced PUFAs accumulation at intermediate frequencies during the stationary growth phase. High-frequency stimulation (1 MHz) induced notable increases in SFAs and a concomitant reduction in UFAs, likely reflecting stress-induced lipid rigidification.

Our findings indicate that AC stimulation can be used to influence FAs saturation patterns. For example, higher SFAs and MUFAs contents may improve biodiesel stability, while PUFAs enrichment, as seen at 1 MHz, could be valuable for biomedical applications.

### FTIR spectral analysis of SCOs

FTIR spectra of lipid extracts from both organisms displayed characteristic lipid-associated vibrational bands such as CH₂ stretching (~ 2920–2850 cm⁻^1^) and ester C = O (~ 1740 cm⁻^1^) were observed [46, 47]. Although qualitative frequency-dependent variations were observed in band intensities and peak sharpness, these trends primarily supported the compositional changes identified by GC–MS. Detailed FTIR spectra, peak assignments, and extended interpretation for all frequencies and time points are provided in the Supplementary Information (Figures S5–S8).

### TEM imaging: ultrastructural alterations

#### TEM imaging: ultrastructural alterations of *Rhodotorula* sp. at 18 h exposure

**Fig. 2:**
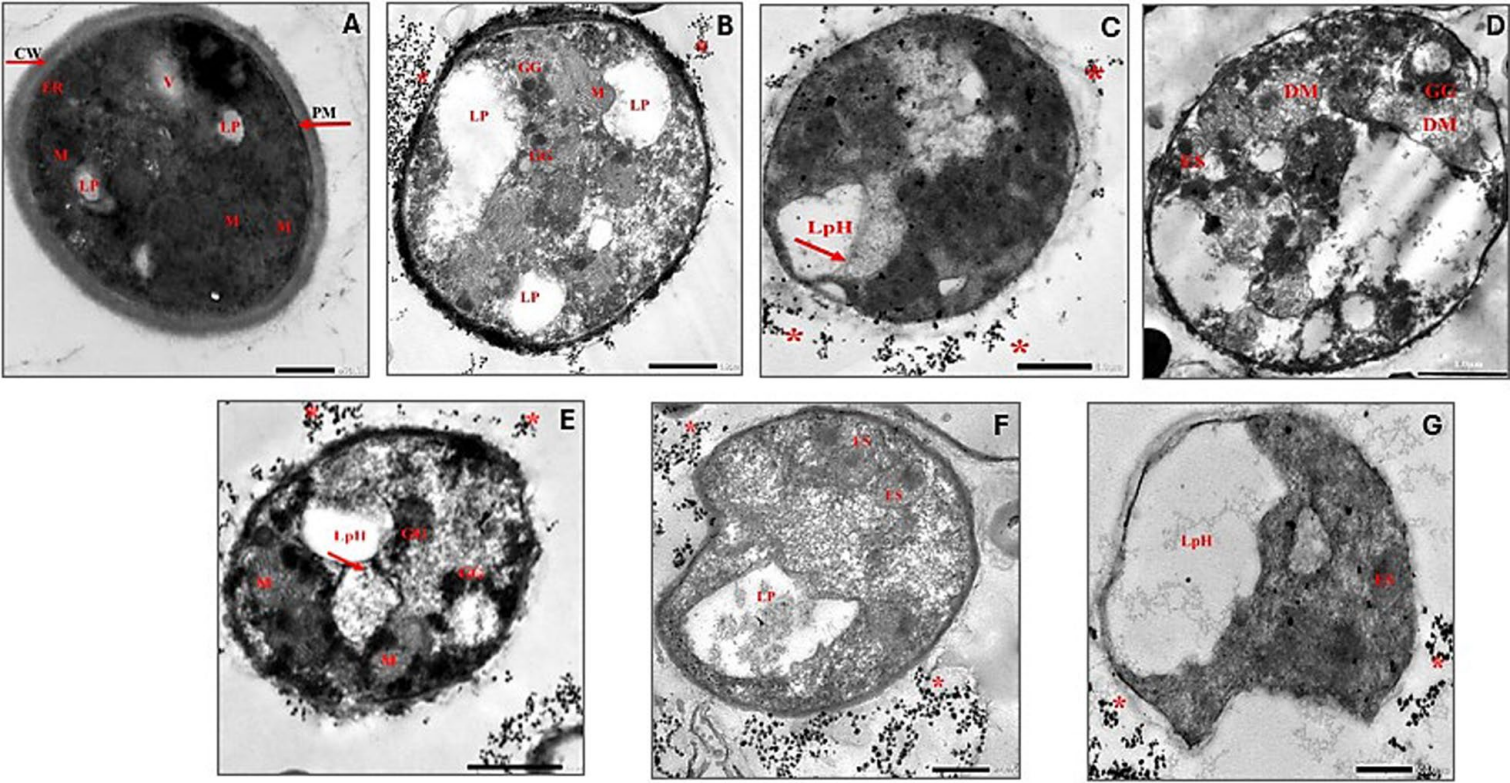
Ultrastructure studies of *Rhodotorula* sp. at 18 h exposure to various AC-regimes. **A** Untreated control, **B** Cells exposed to 100 Hz, **C** and **D** Cells exposed to 1 kHz, **E** Cells exposed to 10 kHz, **F** Cells exposed to 100 kHz, and **G** Cells exposed to 1 MHz. CW: cell wall, PM: periplasmic membrane, ER: endoplasmic reticulum, V: vacuole, M: mitochondria, LP: lipid droplets, GG: glycogen granules, LpH: lipophagy, DM: damaged mitochondria, ES: endosome, P: peroxisome, stars: ruptured particles from mucilaginous capsule

**Fig. 3:**
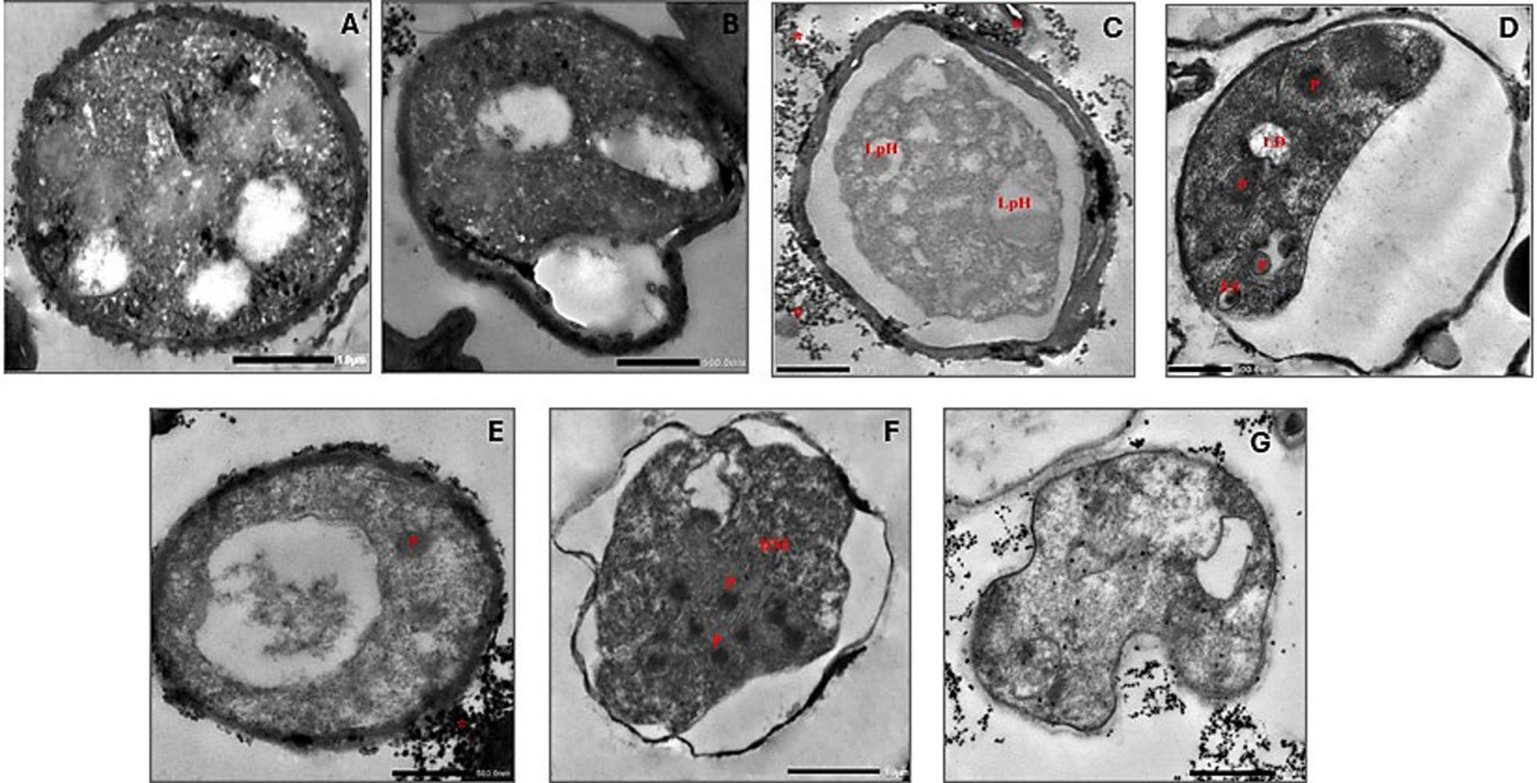
Ultrastructure studies of stationally-growing *Rhodotorula* sp. at 48 h exposure to various AC-regimes. **A** Untreated control, **B** Cells exposed to 100 Hz, **C** Cells exposed to 1 kHz, **D** Cells exposed to 10 kHz, **E** Cells exposed to 100 Hz, **F** and **G** Cells exposed to 1 MHz. CW: cell wall, PM: periplasmic membrane, ER: endoplasmic reticulum, V: vacuole, M: mitochondria, LP: lipid droplets, GG: glycogen granules, LpH: lipophagy, DM: damaged mitochondria, ES: endosome, P: peroxisome, stars: ruptured particles from mucilaginous capsule

The cellular ultrastructures of the oleaginous strain *Rhodotorula* sp. were analyzed and visualized using TEM at two time points during yeast culture: the exponential phase (18 h) and the stationary phase (48 h) under different conditions of AC exposure (Figs. 2 and 3). This was done to observe the overall morphological changes and adaptation behavior of the strain, as well as the formation of SCOs or lipids, indicating lipogenesis under AC influence. Initially, the control cells of *Rhodotorula* sp. (Figs. 2-A and 3-A) displayed the usual features and architecture of eukaryotic cells. In general, its cells retained their healthy ellipsoid to spherical shapes with well-defined cell walls, periplasmic membrane and highly electron-dense cytoplasm contained several dispersed intracellular organelles such as rough endoplasmic reticulum, lamellar mitochondria, vacuole, scattered tiny dark glycogen granules, and small size irregular lipid bodies, which are recognized through their amorphous non-electron-dense content, as described by Marini et al. [48]

However, upon exposure to AC, some variations could be easily observed, which depended on the AC frequencies. Via exposure to 100 Hz (Fig. 2B), the cell wall thickness decreased, and lipid droplets increased in both size and number, indicated by the widening of electron-lucent spaces. That was accompanied by more dispersion of glycogen bodies and mitochondria that seemed intact, thereby reflecting the increase of the AC stressful effect. On the contrary, it was reported previously that *Saccharomyces pastorianus* CGMCC had a cell wall thickness increase from 119.58 nm to 150.96 nm upon exposure to osmotic pressure, as a stress factor, attributing this action to the protective strategy followed by the yeast cell to tolerate osmotic stress [49].

At 1 kHz, the cell wall thickness lessened significantly, despite retaining the cell its morphology, while the electron-lucent areas of the cells increased in size with evidence of the fusion between vacuoles and oil droplets. Such a phenomenon, called autophagy or lipophagy, wherein their enlargement occupied a considerable portion of the cytoplasm (Fig. 2C and D). Besides, damaged mitochondria were also observed. Intriguingly, Kieliszek et al. pointed out the capability of yeast cells to retain their morphological features with stiff structure under stress even when the cell wall was destroyed, which synchronized with our results [54].

However, treatment at 10 kHz induced also autophagy with intensive appearance of peroxisomes and glycogen granules (Fig. 2E). While elevating AC frequencies to 100 kHz and 1 M Hz resulted in deformation of the cells with thinner cell wall and less dense cytoplasm, besides, damaged organelles and the emergence of endosomes with signs of internalization, docking and fusion of lipid droplets with vacuole revealing again the autophagy phenomena (Fig. 2F, and G, respectively). Notably, highly electron-opaque particles were detected scattered surrounding to the cells of *Rhodotorula* sp., which could be attributed to ruptured mucilaginous capsule layer by the action of AC (referred as stars-Fig. 2).

Actually, it is plausible to unveil the cellular importance of autophagy process. It plays a central role in cellular lipid homeostasis by recycling intracellular components and redirecting carbon and energy toward storage pathways when cells experience stress. In oleaginous fungi and yeasts, stress-induced autophagic flux has been shown to enhance triacylglycerol (TAG) accumulation by increasing the availability of acetyl-CoA and NADPH precursors, facilitating lipid droplet biogenesis, and removing damaged organelles that otherwise disrupt lipid metabolism. Autophagy also stabilizes existing lipid droplets by supplying membrane material and regulating lipophagy turnover. The ultrastructural features observed in our TEM analyses such as enlarged lipid bodies and cytoplasmic reorganization under specific AC frequencies, are consistent with these autophagy-linked mechanisms described in prior studies [50–53].

#### TEM imaging: ultrastructural alterations of *Rhodotorula* sp. for 48 h exposure

At 48 h exposure, upon exposure to 100 Hz, the cells appeared distorted in their morphology while maintaining their thick cell wall, dense cytoplasm, and larger lipid bodies (Fig. 3). Notably, higher AC frequencies (i.e., 1, 10, and 100 kHz) caused more stress. The cells could be described as ghost cells exhibiting wider periplasmic spaces, shrinkage or compaction of the cytoplasm, increased cytoplasmic vacuolization, and pyknosis processes. In agreement with our observations, Marini et al. and Rosas-Paz et al. reported large electron-lucent areas inside the cells of *R. mucilaginosa*, attributed to the enlargement of vacuoles and lipid droplets, which occupy a significant portion of the cytoplasm due to stress or nutrient starvation [48, 55]. Additionally, other features such as disintegrated organelles, peroxisomes, and autophagy bodies were also observed (Fig. 3).

Similar features of autolysis and cellular distortion upon exposure of *R. glutinis* and *Yarrowia lipolytica* have been reported to stress factors [54, 56]. Remarkably, 1 MHz induced more cellular distortion with irregularly shaped cells and shrunken cytoplasm owing to protoplast plasmolysis. The signs of autophagy body inside the vacuole and peroxisome were also observed. In this context, Hariri et al. referred to the proximity of lipid droplet location with vacuole, considering that a strategic metabolic mechanism, to facilitate the turnover of lipids in the lipophagy process during prolonged starvation or stress [57].

Based on TEM for *Rhodotorula* sp. At both incubation times, the discernible alterations in cellular ultrastructure coupled with AC-treatment, especially lipid droplets, might be a sign of progressive shifting from lipogenesis to lipid catabolism. Such cellular transition permitted the cells to generate the basal level of energy and sustain adverse conditions caused by nutrient starvation, prolonged incubation, and cell aging. It could be proposed that the lipogenesis process was induced at both examined ages of the yeast (i.e., 18 h and 48 h), with a more intense increase in size and number during 48 h. Upon treatment, the cells exposed to stress conditions behaved differently. Not all cells in the culture possess the same capability to withstand this stress. Some of them were vulnerable cells and might be damaged during treatment. The stress-tolerant cells would cope with these unfavorable conditions and exert adaptive responses by accumulating more lipids/SCOs in the droplets. However, with prolonged incubation (i.e., 48 h), the stress becomes intensive due to nutrient starvation and aging of the cells, which forces the cells to remodulate such metabolism imbalance and utilize storage materials (i.e., lipids and glycogen granules) efficiently, to maintain energy demand [55].

#### TEM imaging: ultrastructural alterations of *A. flavus* at 18 h exposure

**Fig. 4:**
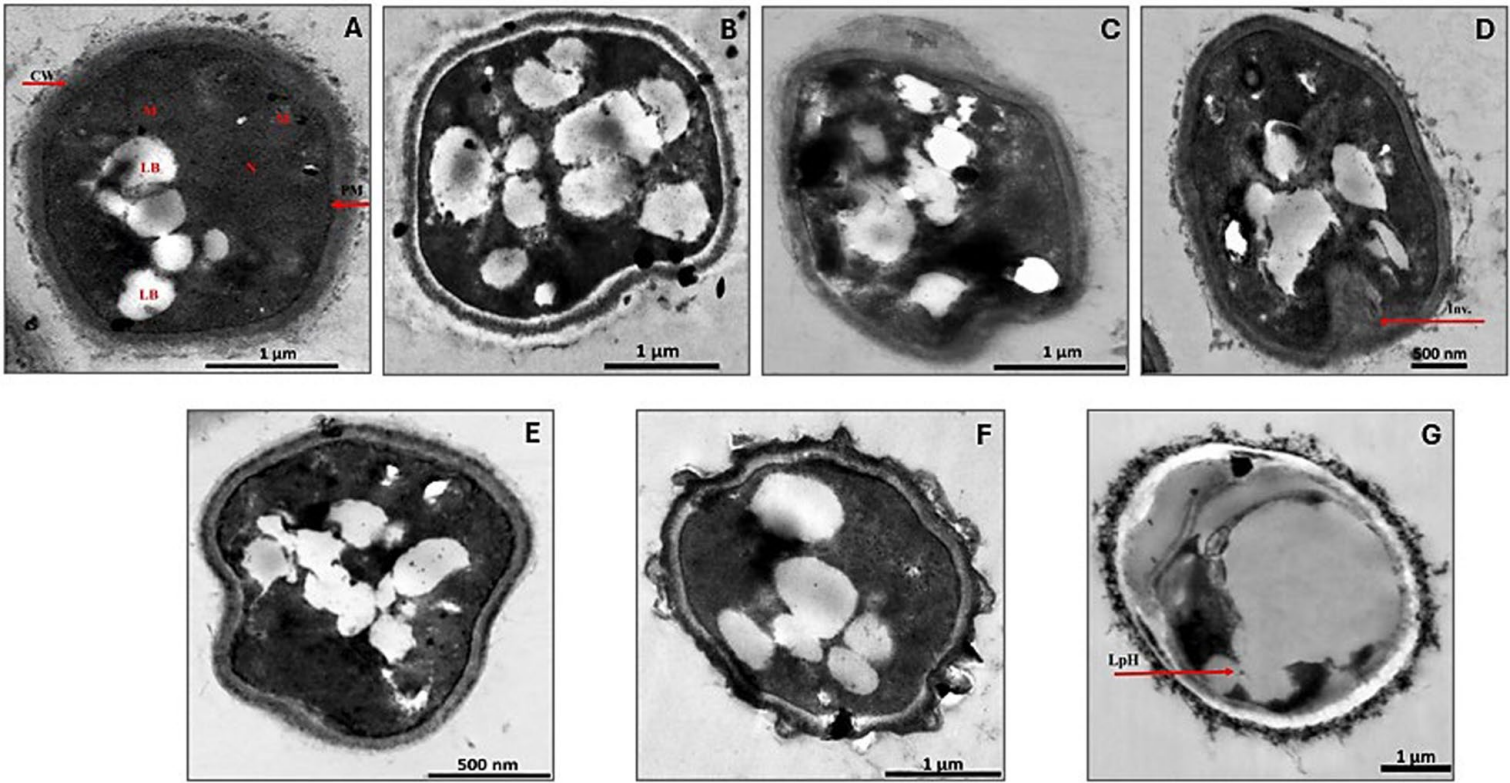
Ultrastructure studies of *A. flavus* at 18 h exposure to various AC-regimes. **A** Untreated control, **B** Cells exposed to 100 Hz, **C** Cells exposed to 1 kHz, **D** Cells exposed to 10 kHz, **E** Cells exposed to 100 Hz, **F** and **G** Cells exposed to 1 MHz. CW: cell wall, PM: periplasmic membrane, N: nucleus, M: mitochondria, LB: lipid bodies, Inv.: plasma membrane invagination, LpH: lipophagy

**Fig. 5:**
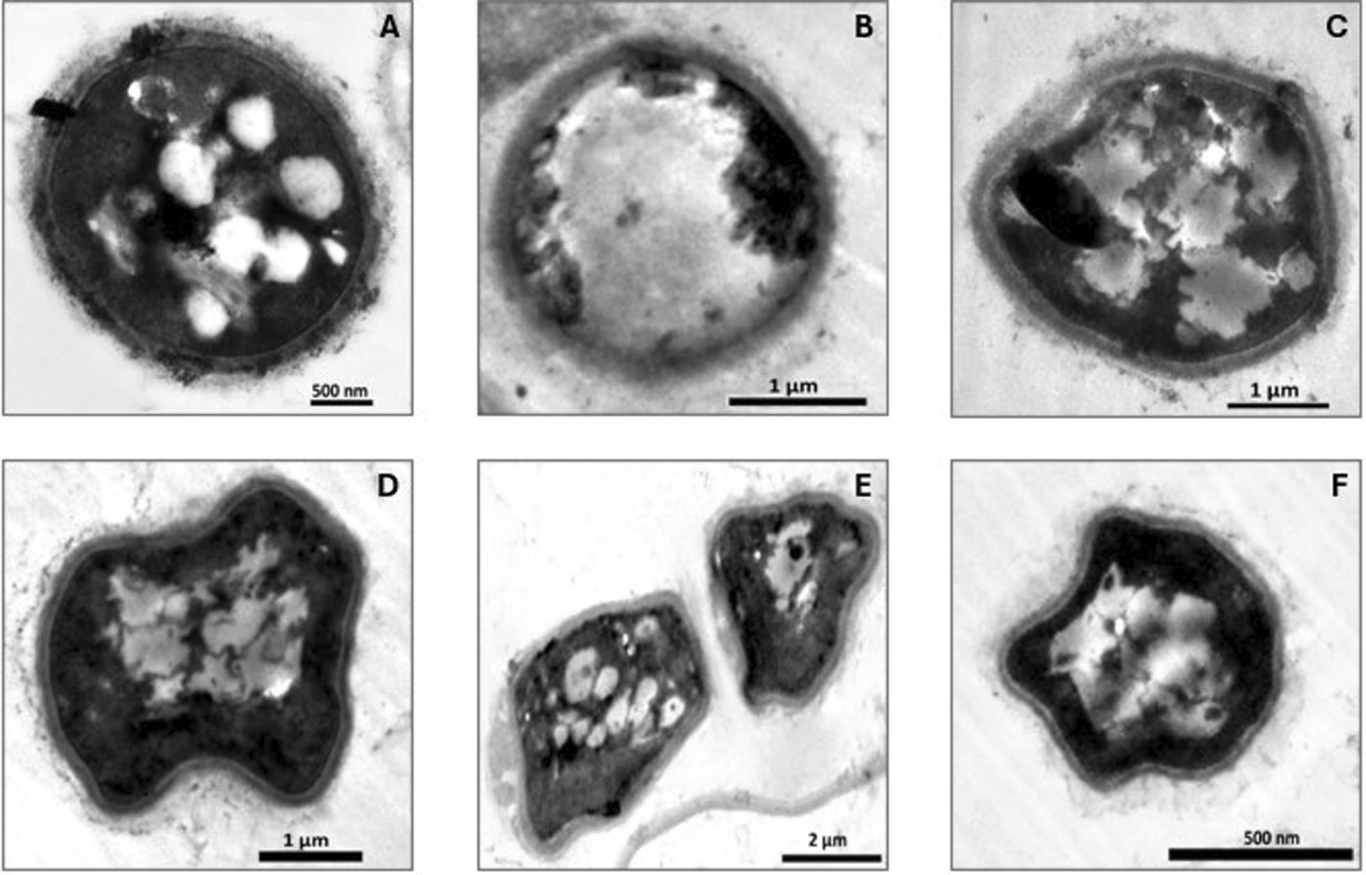
Ultrastructure observations of *A. flavus* at 48 h exposure to various AC-regimes. **A** Untreated control, **B** Cells exposed to 100 Hz, **C** Cells exposed to 1 kHz, **D** Cells exposed to 10 kHz, **E** Cells exposed to 100 Hz, **F** Cells exposed to 1 MHz

Our study involved examining the ultrastructural variation in *A. flavus* pellets when subjected to SCOs accumulation conditions under different AC-regime treatments at 18 h exposure and 48 h exposure (Figs. 4 and 5). Control *A. flavus* cells were able to grow in their regular plump, round, or slightly elliptical morphology with normal dimensions of 3.6 ± 0.8 µm length and 2.4 ± 0.6 µm width. The normal cells were intact and clearly coated with a smooth, thick, uniform cell wall that was thoroughly enclosed by an observable fiber layer. Besides, the plasma membrane appeared healthy, integrated, and unfolded in full uniformity over all cell areas. Meanwhile, dense cytoplasm containing dispersed distribution organelles of nuclei and mitochondria was vividly displayed (Fig. 4A). Notably, several lipid / SCOs bodies were visible in different sizes in the cytoplasm, in the range of 0.24–0.53 µm, as amorphous non-electron-dense spheres. Likewise, such normal features and morphological structure were commonly reported in different species of *Aspergillus* [58].

Upon treatment with 100 Hz, the cells continued to tolerate adverse conditions, which was displayed clearly through maintaining their intact spherical shape but with slight deterioration, while keeping intact thick cell wall, plasma membrane, and dense cytoplasm with an intensive increase in lipid / SCOs bodies’ number and size. Remarkably, such lipid/SCOs bodies appeared irregular and pre-fused together, reaching 0.27–1.3 µm in size, and occupied more than two-thirds of the cellular area (Fig. 4B).

Our TEM results and appearance of lipid/SCOs bodies were harmonized with those obtained and depicted previously by other authors [59]. Meanwhile, more cell deformation was displayed at AC-exposure frequencies of 1, 10, and 100 kHz, despite whole uniformity of cell wall, plasma membrane, and cytosol (Fig. 4C, D, and E). Nonetheless, a sign of a linear deep plasma membrane invagination that reached 700 nm in length with a diameter of 377 nm was detected, especially after 10 kHz exposure (Fig. 4D). In agreement with our findings, the same feature of plasma membrane folding, detachment from the cell wall, and the formation of projections upon exposing the examined fungal cells to stress conditions [48], such plasma membrane folds inward, were deemed as a common response exerted by *Aspergillus* species against exposure to antifungal compounds [60].

Whereas, the exposure to 1 MHz of AC was considered the most stressful dose, represented by less thickening of the cell wall with rough fuzzy appearance, less dense cytoplasm, damaged cytosolic organelles, evacuation of cellular content, and lipophagy (Fig. 4F and 4G). Intriguingly, lipophagy, as a sort of autophagy, is essential for extended recycling of cellular resources during lipid accumulation [61]. This lipophagy phenomenon is a vital metabolic process followed by the eukaryotic cells under stress to sustain viability [56]. The existence of lipid bodies in proximity to the vacuole facilitates the turnover of lipids in the lipophagy process as a response to prolonged incubation, nutrient starvation, and unfavorable conditions in the cell's milieu [57], which is consistent with our results (Fig. 4G).

#### TEM imaging: ultrastructural alterations of *A. flavus* for 48 h exposure

Regarding the control of lipogenic *A. flavus* pellets cells, at the stationary phase (48 h), the cells displayed a typical regular spherical shape surrounded by a rigid multilayer cell wall and plasma membrane with dense cytoplasm, nuclei, and mitochondria with their distinguishable cristae. Notably, more lipid/ SCOs bodies were observed in this stage than those detected in exponentially-growing cells (i.e., 18 h) (Fig. 5A). Antunes et al. reported that *R. glutinis* cells taken during the exponential phase contained just a few lipoidal inclusions, but they expanded in quantity and size to the point where they filled the majority of the cytoplasm compartments in cells retrieved during the stationary phase [62]. The treated senescent fungal cell at 100 Hz displayed the exact observation concerning lipid/SCOs bodies, wherein, they increased in number, varied in shapes, enlarged in size, and fused till filling most of the cytoplasm compartment (Fig. 5B).

Meanwhile, uplifting the exposure dose to 1, 10, 100 kHz and even 1 MHz, induced cell-shape abnormality, which was recognized by losing of their regularity relative to the untreated control (Figs. 5C–F), with slight plasma membrane invagination, without losing membrane integrity, smooth appearance and without any detachment from cell wall. Nevertheless, the cells showed obvious capability to maintain their dense multilayer cell wall, dense cytoplasm without any shrinkage, and with other unrecognized cytosolic organelles owing to filling the cytosolic compartment by lipid/SCOs bodies. In the same context, Zhang et al. reported that *A. niger* cells followed an intelligent mechanism by changing the cell morphology and its specific surface area, to tolerate harsh conditions while maintaining their active metabolic growth and energetic behavior till cell apoptosis [63].

The ultrastructural analysis of *A. flavus* revealed distinct temporal adaptations to AC electrical stimulation between the exponential (18 h) and stationary (48 h) growth phases. At 18 h, cells maintained generally intact morphology with well-defined, thick cell walls and plasma membranes, alongside increased accumulation of lipid/SCO bodies, especially at low to mid frequencies (100 Hz to 10 kHz). Membrane invaginations and localized lipophagy were evident, indicative of early adaptive responses facilitating lipid turnover under moderate stress. In contrast, by 48 h, fungal cells exhibited more pronounced morphological alterations, including irregular cell shapes, roughened and thinner cell walls particularly at high frequencies (100 kHz and 1 MHz), and extensive lipid body expansion that occupied most of the cytoplasm. Despite these changes, plasma membrane integrity was largely preserved, reflecting a strategic remodeling aimed to maintain cellular viability during prolonged stress and nutrient limitation. The increased prevalence of lipophagy and autophagic features at 48 h exposure further suggests a metabolic shift from lipid accumulation toward lipid catabolism to sustain energy homeostasis during stationary phase. Overall, *A. flavus* displays a dynamic ultrastructural transition from growth-phase lipid storage to stress-associated remodeling and resource recycling, modulated by both AC frequency and cells age.

#### Comparison between ultrastructural analysis and interpretation in *Rhodotorula* sp. and *A. flavus*

Our TEM revealed that both *Rhodotorula* sp. and *A. flavus* undergo pronounced ultrastructural remodeling in response to AC stimulation, with effects varying by exposure frequency and growth phase. At 18 h, *Rhodotorula* sp. cells exhibited increased lipid droplet formation, autophagic features including vacuole-lipid droplet fusion (lipophagy), and moderate organelle damage that intensified with increasing AC frequency. Similarly, *A. flavus* maintained intact morphology under low-frequency exposure (100 Hz). Semi-quantitative analysis of TEM micrographs (ImageJ-based) was performed using calibrated scale bars to estimate apparent cross-sectional lipid droplet diameters (n = 15–20 droplets per condition). Measurement of representative droplets in *Rhodotorula* sp (18 h exposure) revealed an increase from 0.26 ± 0.09 µm in control cells to 0.56 ± 0.38 µm under 100 Hz stimulation, corresponding to a 114% increase in mean apparent diameter. Similarly, in *A. flavus* (18 h exposure), the mean droplet diameter increased from 0.34 ± 0.08 µm to 0.62 ± 0.15 µm under the same frequency, representing an 82% enlargement. Although these measurements represent two-dimensional sections and may underestimate true droplet volume, the marked increase in cross-sectional size supports the interpretation that AC stimulation promotes droplet enlargement and fusion events consistent with the enhanced lipid yield.

The results show an enhanced lipid body accumulation and membrane invaginations at mid frequencies (1–100 kHz). The concomitant observation of increased SFAs and plasma membrane invaginations at 10 kHz suggests a coordinated structural–metabolic adaptation. One plausible interpretation is a homeoviscous adaptation response, whereby cells increase membrane saturation to enhance bilayer rigidity in order to counteract oscillatory deformation induced by AC. Repeated polarization–depolarization cycles at mid-range frequencies may impose mechanical stress on the membrane, and increased incorporation of saturated acyl chains would reduce membrane fluidity and stabilize membrane architecture. Thus, the frequency-dependent enrichment of SFAs at 1–10 kHz may represent a protective stiffening mechanism aligned with the ultrastructural changes observed by TEM. High-frequency exposure (1 MHz) induced severe stress in both organisms, manifesting as cell wall thinning, cytoplasmic depletion, organelle damage, and prominent lipophagy, reflecting oxidative and metabolic stress responses.

By prolonged incubation at 48 h, AC treatment led to more severe morphological distortions in both organisms, including ‘ghost cell’ formation, cytoplasmic shrinkage, and pyknosis in *Rhodotorula* sp., alongside increased lipid droplet size and number. In *A. flavus*, lipid bodies dominated the cytoplasm, and despite irregular cell shapes at higher frequencies, plasma membrane integrity and multilayered cell walls were largely preserved, suggesting adaptive remodeling to sustain viability.

In the present study, the term “ghost cells” refers to cells exhibiting marked cytoplasmic depletion and reduced electron density, while retaining an identifiable cell wall and plasma membrane outline under TEM. This designation refers to an ultrastructural phenotype rather than a confirmed viability status. The preserved membrane architecture suggests that these cells are not undergoing complete lytic destruction; however, whether this state represents reversible physiological stress adaptation or terminal loss of viability cannot be conclusively determined in the absence of dedicated viability assays. Therefore, the PUFA enrichment observed at 1 MHz may arise from either active stress-adaptive lipid remodeling in surviving cells or from selective persistence of membrane lipid fractions during stress-associated cytoplasmic collapse.

These ultrastructural changes indicate a dynamic balance between lipogenesis and lipid catabolism regulated by AC stimulation. At 18 h, lipid accumulation supports energy storage, while at 48 h, it triggers autophagic turnover to meet cellular energy demands under nutrient limitation and oxidative stress. The observed plasma membrane invaginations and endosome-like structures further highlight complex membrane remodeling processes essential for fungal stress adaptation and SCOs metabolism.

The TEM findings underscore the capability of *Rhodotorula* sp. and *A. flavus* to modulate cellular ultrastructure in a frequency- and growth phase-dependent manner under electrical stimulation, providing morphological evidence for metabolic shifts that correlate with the biochemical and compositional lipid changes observed via GC–MS and FTIR analyses.

Collectively, the findings suggest the presence of three distinct frequency-dependent biological regimes governing lipid metabolism under AC stimulation. Low-frequency exposure (100 Hz–1 kHz) was characterized by enhanced lipid droplet biogenesis, increased total lipid yield, and relative preservation of cellular ultrastructure, indicating a productive activation state compatible with sustained lipid accumulation. Mid-frequency stimulation (10–100 kHz) was associated with membrane rigidification and a pronounced reduction in UFAs, suggesting selective interference with desaturase-mediated lipid modification and representing a potential inhibitory frequency window for unsaturation pathways. In contrast, high-frequency exposure (1 MHz) promoted PUFA enrichment concurrent with cytoplasmic depletion and autophagic features, consistent with a stress-adaptive membrane remodeling response rather than a stable high-yield production phenotype. These observations support the concept that AC frequency functions as a tunable bioelectrical parameter capable of differentially modulating lipid metabolism through coordinated structural, enzymatic, and membrane biophysical mechanisms.

## Conclusions

This study demonstrates that AC frequency modulation is a novel, chemical-free method for controlling both the quantity and composition of microbial oils. In *Rhodotorula* sp. and *A. flavus*, low-frequency stimulation (100 Hz–1 kHz) delivered the highest lipid yields—up to 2.7-fold above controls—while producing a favorable SFAs/MUFAs balance rich in oleic acid and palmitic/stearic acids, ideal for biodiesel stability and cold-flow performance. While exposure to 1 MHz selectively enriched PUFAs, particularly omega-6 linoleic acid, relevant for nutraceutical and biomedical applications. Mid-range frequencies (10–100 kHz) favored MUFAs accumulation, especially oleic acid, supporting uses in both biofuels and heart-healthy dietary formulations. Ultrastructural analysis linked these biochemical shifts to frequency-specific morphological adaptations, including lipid droplet expansion, membrane remodeling, and autophagic turnover. These results highlight AC frequency as a tunable “metabolic switch” to target lipid profiles for specific industrial and health-related applications. Future integration of lipidomics, metabolomics, and proteomics will be key for mapping regulatory pathways and scaling AC-driven lipid engineering for commercial production.

## Supplementary Information

Additional file 1.

## Data Availability

All data are included in the manuscript and the supplementary files.
