# Supplementary material for: Frequency-dependent modulation of functional single cell oils in Rhodotorula sp. and Aspergillus flavus under alternating current stimulation
Source: Biotechnol Biofuels Bioprod. 2026 Apr 2;19:34. doi: 10.1186/s13068-026-02757-3 (PMC13064357; doi:10.1186/s13068-026-02757-3)
Supplement: Supplementary file 1 — Additional file 1. [file 13068_2026_2757_MOESM1_ESM.docx]

**Frequency-Dependent Modulation of Functional Single Cell Oils in *Rhodotorula* sp. and *Aspergillus flavus* under Alternating Current Stimulation**

**Hadeel El-Shall ^1*^, Afaf A. Gliwan ^2^, Mamdouh M. Shawki ^2*^, Marwa M. Eltarahony ^1*^, and Moataz M. Fahmy ^2^**

^1^ Environmental Biotechnology Department, Genetic Engineering and Biotechnology Research Institute, City of Scientific Research and Technological Applications (SRTA-City), Alexandria, Egypt. hadeel.elshall28@gmail.com and m_eltarahony@yahoo.com.

^2^ Medical Biophysics Department, Medical Research Institute, Alexandria University, Alexandria, Egypt. afaf.gliwan@gmail.com, mamdouh971@hotmail.com, and moataz.m.fahmy@alexu.edu.eg.

*** Correspondence:**

**Marwa M. Eltarahony:** m_eltarahony@yahoo.com

**Hadeel El-Shall :** hadeel.elshall28@gmail.com

**Mamdouh M. Shawki:** [mamdouh971@hotmail.com](mailto:mamdouh971@hotmail.com)

**Table S1. Full GC-MS data, including RT and molecular formula identified in *Rhodotorula* sp. Cells after 18 h exposure of Electrical Stimulation (Area %)**

| Compound Name | Common Name | RT | Type of FA | Molecular Formula | Control Group | 100 Hz | 1 kHz | 10 kHz | 100 kHz | 1 MHz |
| --- | --- | --- | --- | --- | --- | --- | --- | --- | --- | --- |
| Hexadecanoic acid | Palmitic acid | 23.21 | SFA | C16H32O2 | 11.54 | 15.28 | 14.85 | 12.20 | 13.59 | 14.16 |
| Hexadecanoic acid,  methyl ester | Methyl palmitate | 22.52 | SFA | C17H34O2 | – | – | – | 0.81 | 0.93 | – |
| 9-Octadecenoic acid | Oleic acid  (omega-9) | 25.94 | MUFA | C18H34O2 | 23.65 | 22.47 | 26.33 | 28.62 | 27.14 | 20.26 |
| 9-Octadecenoic acid,  methyl ester | Methyl oleate | 25.98 | MUFA | C19H36O2 | – | – | – | 1.29 | – | 2.42 |
| 9,12-Octadecadienoic acid | Linoleic acid  (omega-6) | 25.73 | PUFA | C18H32O2 | 0.18 | 0.24 | 0.35 | 0.25 | 0.26 | 11.26 |
| 9,12-Octadecadienoic  acid, methyl ester | Methyl linoleate | 25.73 | PUFA | C19H34O2 | – | – | – | 0.94 | – | – |
| Octadecanoic acid | Stearic acid | 20.92 | SFA | C18H36O2 | 6.70 | 5.15 | 4.94 | 2.19 | 3.08 | 2.76 |
| Octadecanoic acid,  methyl ester | Methyl stearate | 26.37 | SFA | \| C19H38O2 \| \| --- \| | – | – | – | 0.78 | 1.21 | 2.38 |
| 9,15-Octadecadienoic acid | – | 28.72 | PUFA | C18H32O2 | – | – | – | – | – | 0.52 |
| 9-Hexadecenoic acid | Palmitoleic acid | 23.98 | MUFA | C16H30O2 | 1.24 | 0.82 | 0.66 | 0.33 | 0.42 | 0.79 |
| Tetradecanoic acid | Myristic acid | 19.62 | SFA | C14H28O2 | 0.54 | 0.86 | 0.71 | 0.75 | 0.82 | 0.63 |
| Octadecanoic acid, 9,10-dihydroxy-, methyl ester | Methyl 9,10-dihydroxystearate | 30.31 | SFA | C19H38O4 | – | – | – | – | – | 0.82 |

**Table S2. Full GC-MS data, including RT and molecular formula identified in *Rhodotorula* sp. Cells after 48 h exposure of Electrical Stimulation (Area %)**

| Compound Name | Common Name | RT |  | Type of FA | Molecular  Formula | Control Group | 100 Hz | 1 kHz | 10 kHz | 100 kHz | 1 MHz |
| --- | --- | --- | --- | --- | --- | --- | --- | --- | --- | --- | --- |
| Hexadecanoic acid | Palmitic acid | 23.16 |  | SFA | C16H32O2 | 11.9 | 13.38 | 13.05 | 13.78 | 6.44 | 19.45 |
| 9-Octadecenoic acid | Oleic acid  (omega-9) | 25.88 |  | MUFA | C18H34O2 | 24.2 | 13.72 | 11.23 | 9.21 | 6.08 | 12.57 |
| 9-Octadecenoic acid,  methyl ester | Methyl oleate | 25.97 |  | MUFA | C19H36O2 | – | 0.50 | – | 0.82 | – | 1.39 |
| 9,12-Octadecadienoic acid | Linoleic acid  (omega-6) | 25.72 |  | PUFA | C18H32O2 | 0.18 | 7.58 | 6.24 | 4.83 | 3.03 | 19.18 |
| Octadecanoic acid | Stearic acid | 26.35 |  | SFA | C18H36O2 | 6.7 | 2.7 | 3.3 | 2.68 | 1.09 | 3.28 |
| Tetradecanoic acid | Myristic acid | 19.62 |  | SFA | C14H28O2 | 0.54 | 0.72 | 0.45 | – | – | – |
| Octadecanoic acid, 9,10-dihydroxy-,  methyl ester | Methyl 9,10-dihydroxystearate | 30.30 |  | SFA | C19H38O4 | 0.82 | 0.66 | – | 0.37 | – | – |

**Table S3. Full GC-MS data, including RT and molecular formula identified in *A. flavus* Cells after 18 h exposure of Electrical Stimulation (Area %)**

| Compound Name | Common Name | RT | Type of FA | Molecular Formula | Control | 100 Hz | 1 kHz | 10 kHz | 100 kHz | 1 MHz |
| --- | --- | --- | --- | --- | --- | --- | --- | --- | --- | --- |
| Hexadecanoic acid, methyl ester | Methyl palmitate | 23.05 | SFA | C₁₇H₃₄O₂ | 14.90 | – | 20.56 | 24.22 | 6.44 | 15.20 |
| 9-Octadecenoic acid, methyl ester | Methyl oleate | 25.73 | MUFA | C₁₉H₃₆O₂ | 7.02 | 8.68 | – | – | 4.14 | 7.48 |
| 11-Octadecenoic acid, methyl ester | Methyl vaccenate | 25.73 | MUFA | C₁₉H₃₆O₂ | – | – | 10.93 | 10.97 | – | – |
| 9,12-Octadecadienoic acid, methyl ester | Methyl linoleate | 25.58 | PUFA | C₁₉H₃₄O₂ | 7.15 | 1.74 | 3.16 | 1.75 | 3.03 | 3.17 |
| 11,14-Eicosadienoic acid, methyl ester | Methyl eicosadienoate | 27.01 | PUFA | C₂₁H₃₈O₂ | – | 0.36 | 0.41 | – | – | 0.30 |
| Octadecanoic acid, methyl ester | Methyl stearate | 26.22 | SFA | C₁₉H₃₈O₂ | 9.33 | 11.50 | 15.48 | 15.86 | 1.09 | 11.49 |
| Tetradecanoic acid, methyl ester | Methyl myristate | 19.50 | SFA | C₁₅H₃₀O₂ | – | 1.52 | 0.97 | 0.88 | – | 1.27 |
| Pentadecanoic acid, methyl ester | – | 21.29 | SFA | C₁₆H₃₂O₂ | – | 1.00 | 0.83 | 0.87 | – | 0.88 |
| Heptadecanoic acid, methyl ester | Methyl margarate | 24.64 | SFA | C₁₈H₃₆O₂ | – | 0.78 | – | 0.58 | – | 1.04 |
| Eicosanoic acid, methyl ester | Methyl arachidate | 29.14 | SFA | C₂₁H₄₂O₂ | – | 0.67 | 0.70 | – | – | – |
| Nonadecanoic acid, methyl ester | – | 31.85 | SFA | C₂₀H₄₀O₂ | – | – | – | – | – | 0.29 |
| Docosanoic acid, methyl ester | Methyl behenate | 31.85 | SFA | C₂₃H₄₆O₂ | 0.23 | 0.26 | – | – | – | – |
| Tetracosanoic acid, methyl ester | Methyl lignocerate | 33.97 | SFA | C₂₅H₅₀O₂ | – | 0.62 | – | 0.58 | – | 0.54 |

**Table S4. Full GC-MS data, including RT and molecular formula identified in *A. flavus* Cells after 48 h exposure of Electrical Stimulation (Area %)**

| Compound Name | Common Name | RT | Type | Molecular Formula | Control | 100 Hz | 1 kHz | 10 kHz | 100 kHz | 1 MHz |
| --- | --- | --- | --- | --- | --- | --- | --- | --- | --- | --- |
| Hexadecanoic acid, methyl ester | Methyl palmitate | 23.15 | SFA | C₁₇H₃₄O₂ | 18.00 | 21.68 | 14.76 | 12.66 | 21.56 | 25.96 |
| Octadecanoic acid, methyl ester | Methyl stearate | 26.31 | SFA | C₁₉H₃₈O₂ | 10.50 | 15.21 | 11.84 | 7.78 | 18.09 | 18.45 |
| Tetradecanoic acid, methyl ester | Methyl myristate | 19.50 | SFA | C₁₅H₃₀O₂ | 1.00 | 1.45 | 1.66 | 2.53 | 1.04 | 1.28 |
| Pentadecanoic acid, methyl ester | Methyl pentadecanoate | 21.28 | SFA | C₁₆H₃₂O₂ | 1.00 | 1.14 | 0.89 | 0.53 | 0.94 | 0.76 |
| Heptadecanoic acid, methyl ester | Methyl heptadecanoate | 24.64 | SFA | C₁₈H₃₆O₂ | 0.80 | 1.16 | 1.00 | 1.11 | 1.11 | 0.75 |
| Tetracosanoic acid, methyl ester | Methyl tetracosanoate | 33.97 | SFA | C₂₅H₅₀O₂ | 0.50 | 1.14 | 0.62 | 0.61 | 0.64 | 0.49 |
| Eicosanoic acid, methyl ester | Methyl eicosanoate | 29.14 | SFA | C₂₁H₄₂O₂ | 0.60 | 0.91 | 1.00 | 1.13 | 1.13 | — |
| 9-Octadecenoic acid, methyl ester) | Methyl oleate | 25.77 | MUFA | C₁₉H₃₆O₂ | 8.00 | 10.23 | 5.45 | 3.61 | 11.14 | 2.72 |
| 11-Octadecenoic acid, methyl ester | – | 25.83 | MUFA | C₁₉H₃₆O₂ | 6.00 | 15.21 | 5.45 | 3.61 | 11.14 | 0.77 |
| 10-Octadecenoic acid, methyl ester | – | 25.83 | MUFA | C₁₉H₃₆O₂ | 0.50 | 0.85 | — | 0.77 | — | 0.77 |
| 9,12-Octadecadienoic acid | Methyl linoleate | 25.58 | PUFA | C₁₉H₃₄O₂ | 1.00 | 1.90 | 2.18 | 3.22 | 4.16 | 1.95 |
| 7,10-Octadecadienoic acid, methyl ester | – | 27.00 | PUFA | C₁₉H₃₄O₂ | 0.50 | 0.85 | 0.34 | 0.86 | 0.86 | 0.55 |


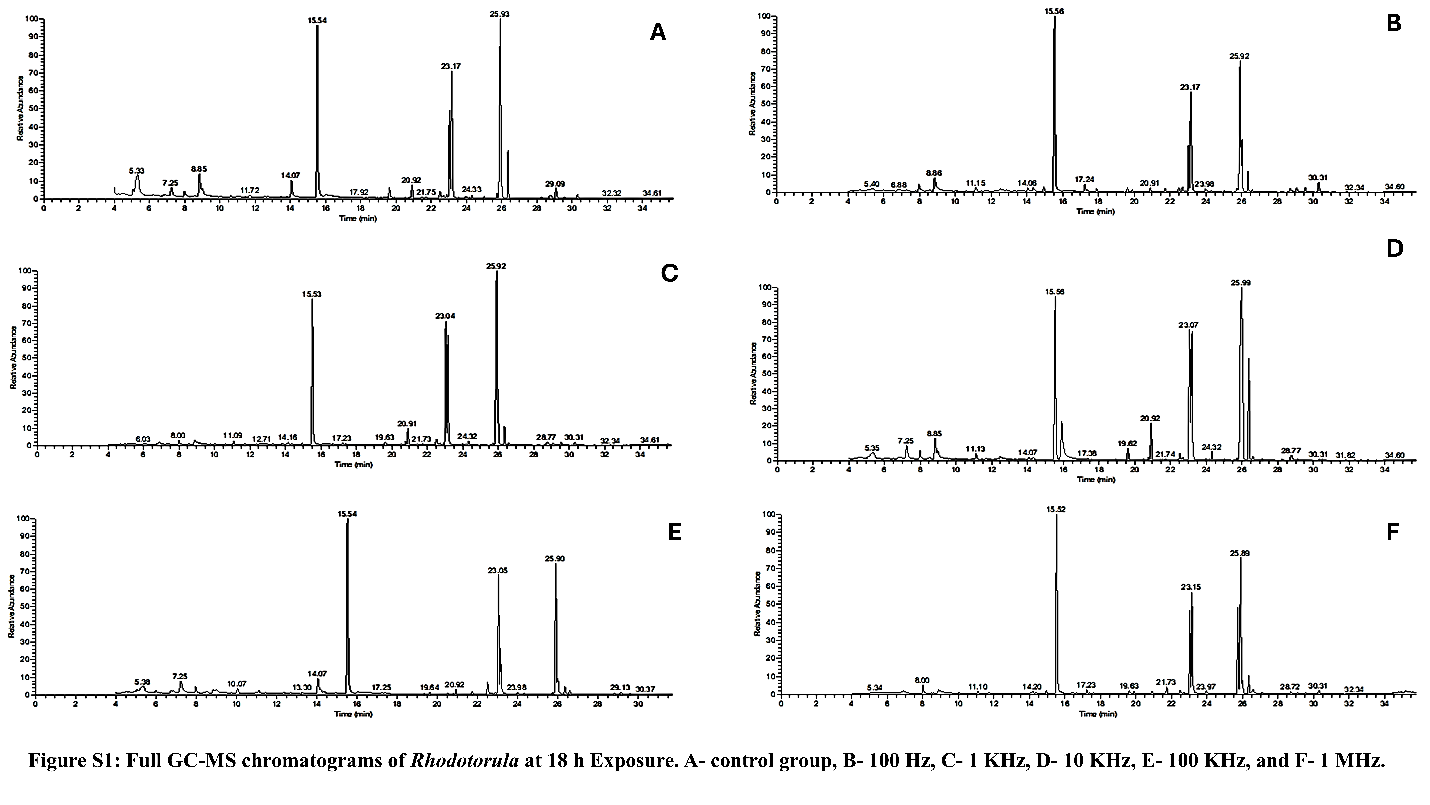


**Figure S1:** Full GC-MS chromatograms of Rhodotorula sp. lipid at 18 h exposure. A-Control group, B-100 Hz, C-1KHz, D-10KHz, E-100 KHz, and F-MHz


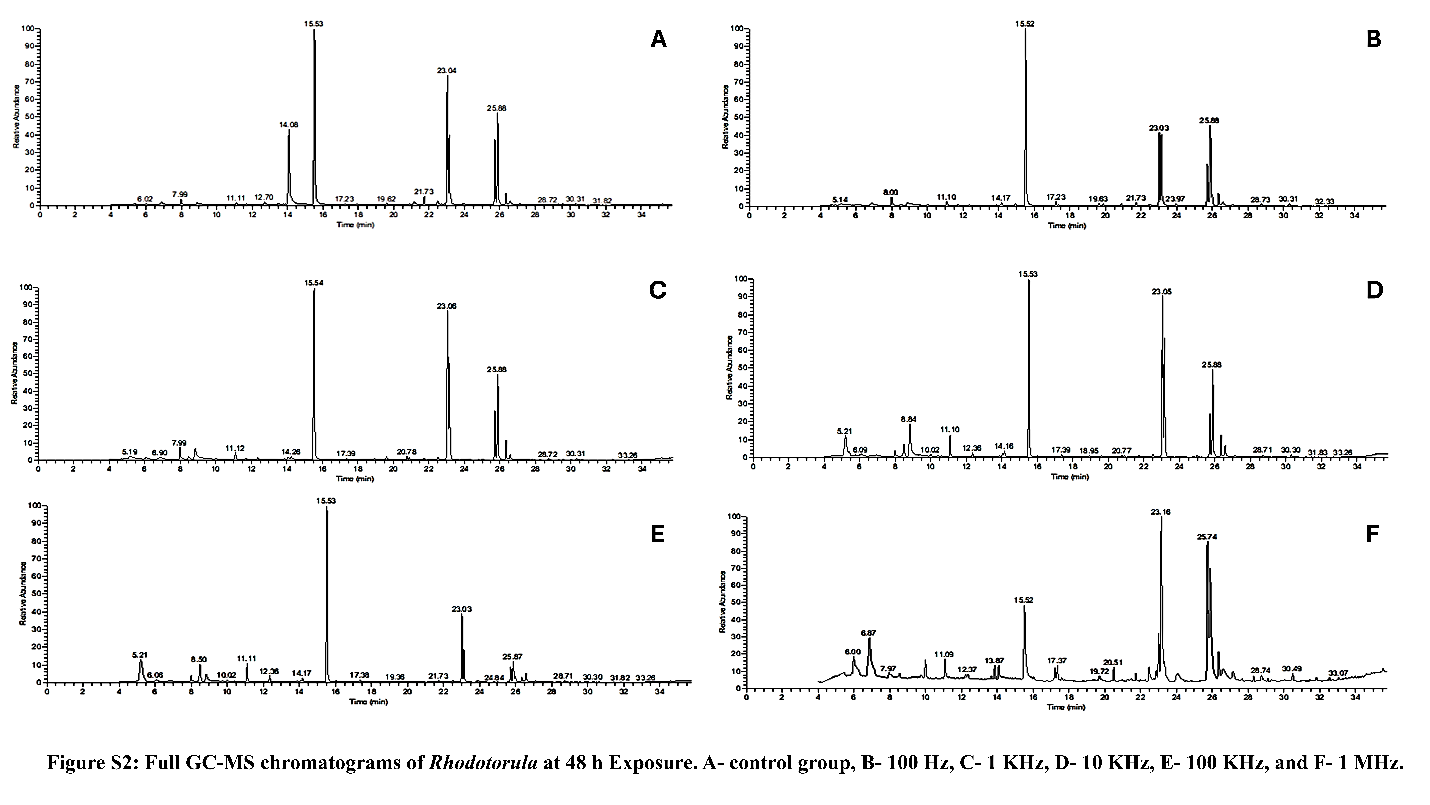


**Figure S2:** Full GC-MS chromatograms of *Rhodotorula* sp. lipid at 48 h exposure. A-Control group, B-100 Hz, C-1KHz, D-10KHz, E-100 KHz, and F-MHz


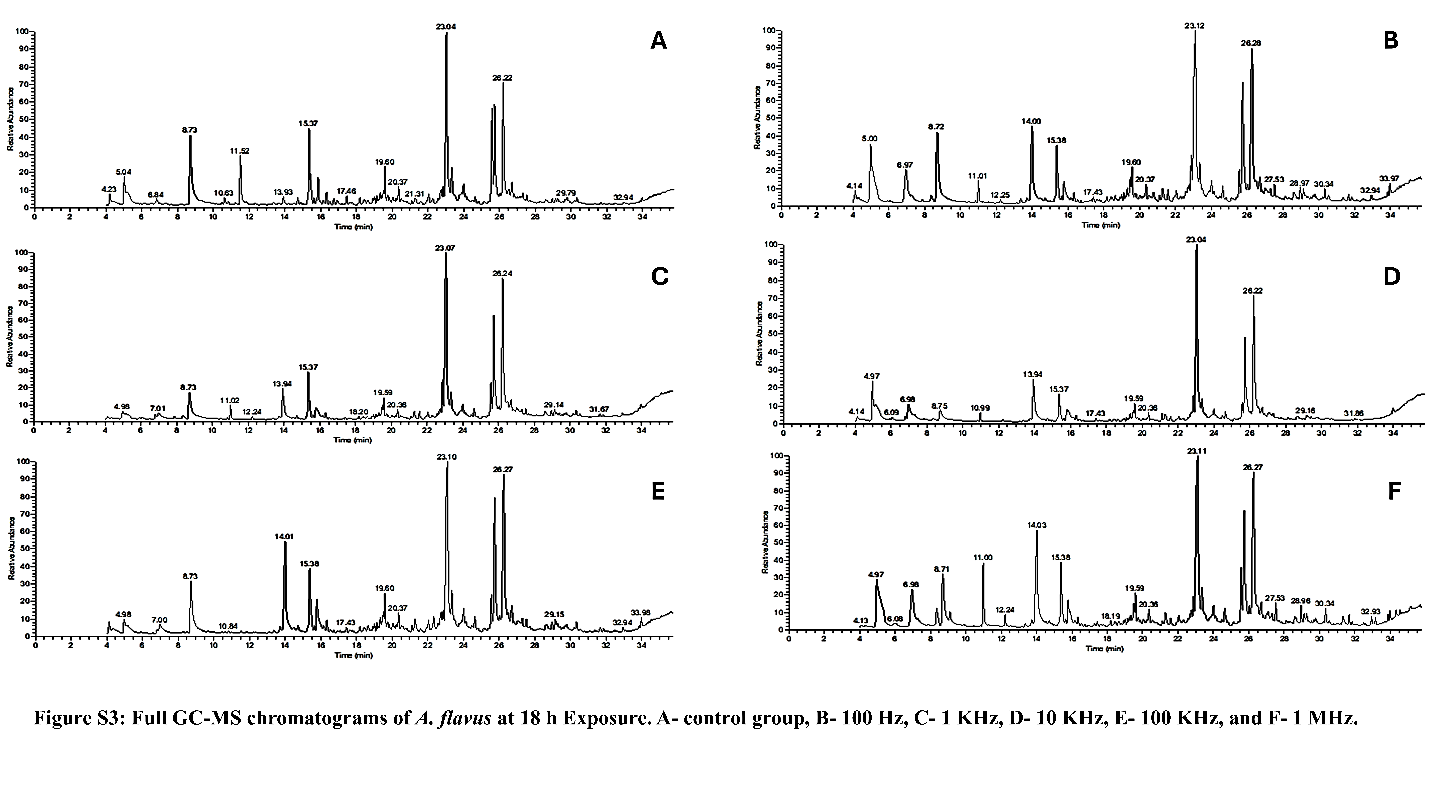


**Figure S3:** Full GC-MS chromatograms of A. flavus lipid at 18 h exposure. A-Control group, B-100 Hz, C-1KHz, D-10KHz, E-100 KHz, and F-MHz


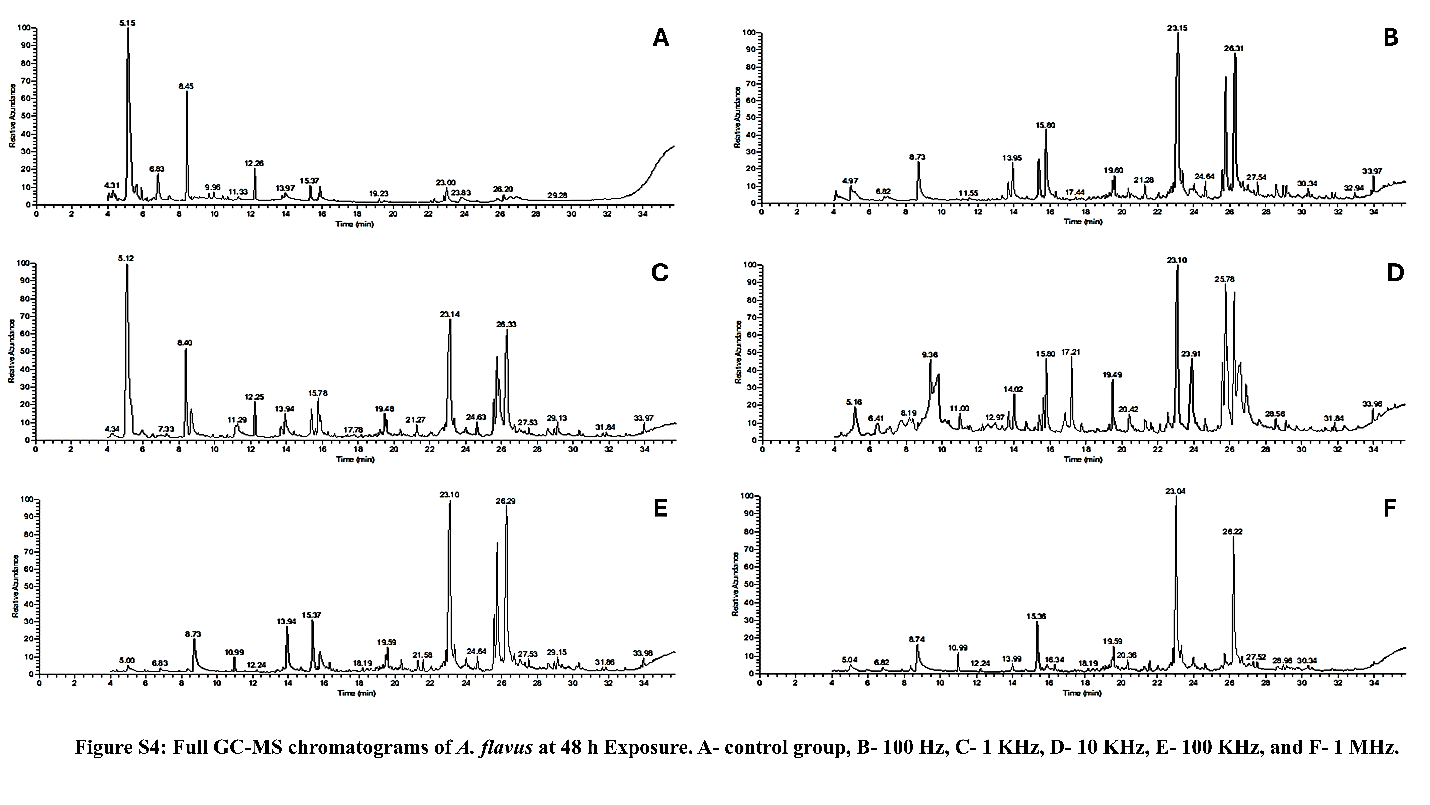


**Figure S4:** Full GC-MS chromatograms of A. flavus at 48 h lipid exposure. A-Control group, B-100 Hz, C-1KHz, D-10KHz, E-100 KHz, and F-MHz


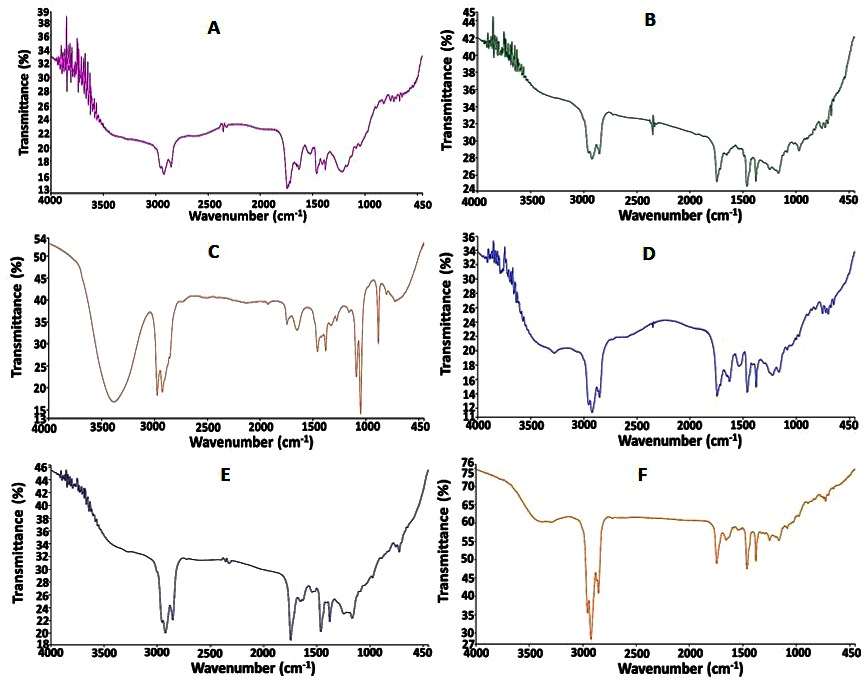


**Figure S5:** FTIR spectra of Rhodotorula sp. lipids at 18 h exposure. A-Control group, B-100 Hz, C-1KHz, D-10KHz, E-100 KHz, and F-MHz

A strong and broad band at 2920–2850 cm⁻¹ corresponds to CH₂/CH₃ stretching vibrations of aliphatic fatty-acid chains. The sharp peak near 1740 cm⁻¹ indicates ester carbonyl (C=O) stretching, confirming the presence of fatty-acid esters. CH₂/CH₃ bending modes appeared at 1465–1375 cm⁻¹, while the 1170–1100 cm⁻¹ region reflected C–O stretching of ester linkages. Frequency-dependent variations were evident, with the 10 kHz sample showing intensified carbonyl and methylene bands, suggesting enhanced lipid accumulation or esterification. In contrast, the 1 MHz group exhibited broader and attenuated peaks, consistent with oxidative or structural alterations in acyl-chain packing.


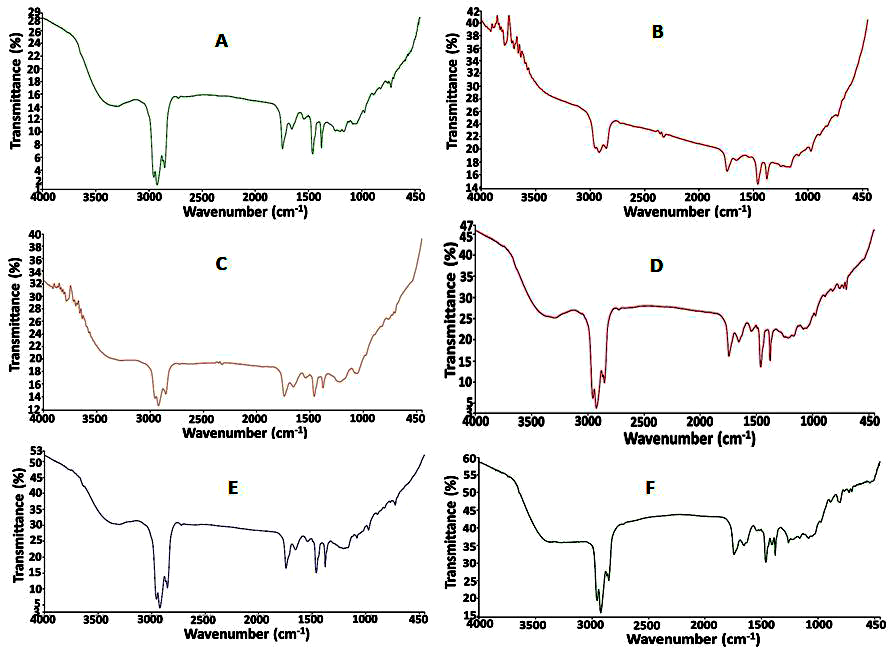


**Figure S6:** FTIR spectra of Rhodotorula sp. lipids at 48 h exposure. A-Control group, B-100 Hz, C-1KHz, D-10KHz, E-100 KHz, and F-MHz

Strong absorption at 2920 and 2850 cm⁻¹ indicates sustained long-chain fatty-acid content across treatments. The ester C=O band (~1740 cm⁻¹) was most pronounced at lower frequencies (100 Hz and 1 kHz), consistent with GC–MS findings showing higher unsaturated lipid fractions under these conditions. Broad O–H stretching (3300–3400 cm⁻¹) weakened relative to 18 h exposure, possibly reflecting reduced free hydroxyl groups through oxidation or esterification. The CH₂/CH₃ bending region (1450–1370 cm⁻¹) showed lower intensity at 1 MHz, consistent with increased saturation. Mid-range frequencies (10–100 kHz) showed more intense C–O/glycerol signals in the fingerprint region (1200–900 cm⁻¹), suggesting active lipid remodeling.


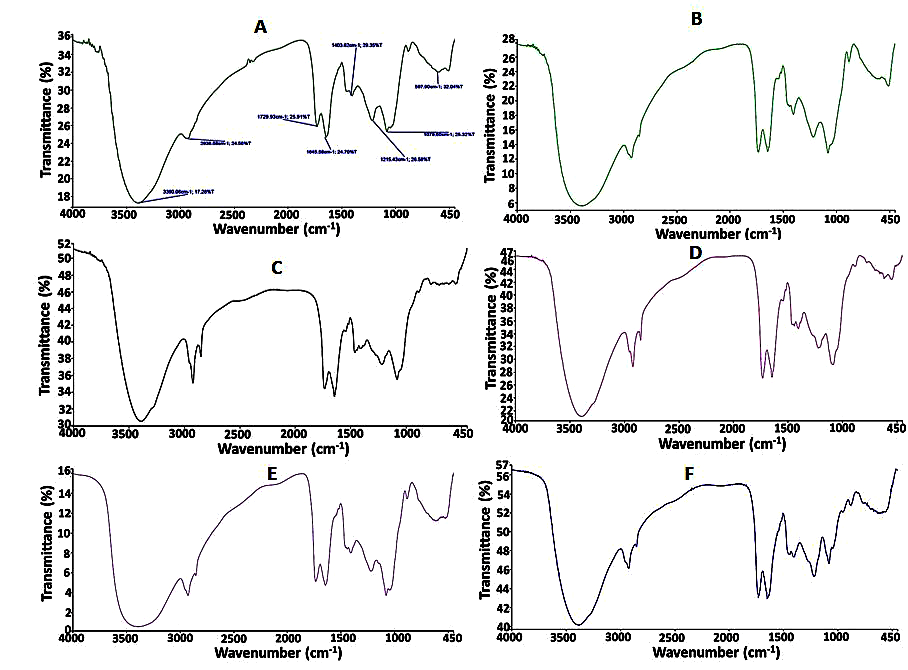


**Figure S7:** FTIR spectra of A. flavus lipids at 18 h exposure. A-Control group, B-100 Hz, C-1KHz, D-10KHz, E-100 KHz, and F-MHz

All groups exhibited a broad, strong band at 3100–2800 cm⁻¹ representing CH₂/CH₃ stretching in long-chain fatty acids. Low-frequency groups (control, 100 Hz, 1 kHz) displayed comparable intensities, with sharper peaks at 1 kHz, consistent with elevated SFA (e.g., methyl palmitate, methyl stearate). A prominent ester carbonyl peak (~1740 cm⁻¹) at 1–10 kHz reflects increased esterified SFAs and MUFAs content. Moderate C=C stretching (~1650 cm⁻¹) was more evident in mid-frequency samples, indicating higher unsaturation. The fingerprint region (1500–900 cm⁻¹) showed sharper and more complex bands at 10 and 100 kHz, suggesting greater lipid diversity or minor PUFAs contributions. The 1 MHz group showed smoother spectra with fewer unsaturation-related bands, consistent with reduced PUFAs content and early oxidative effects.


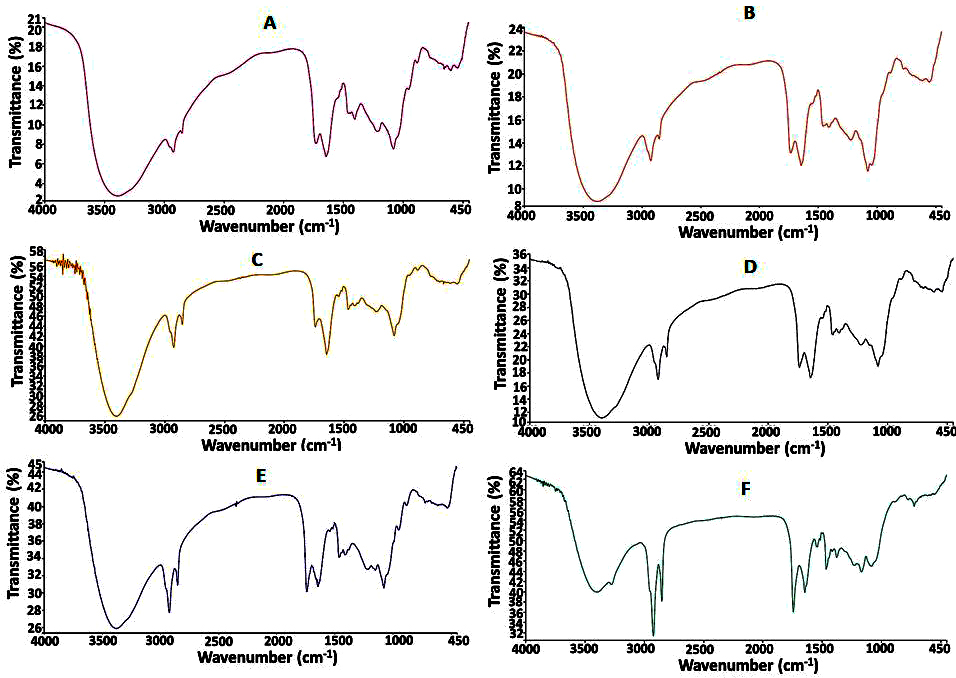


**Figure S8:** FTIR spectra of A. flavus lipids at 48 h exposure. A-Control group, B-100 Hz, C-1KHz, D-10KHz, E-100 KHz, and F-MHz

The control sample exhibited broad O–H stretching near 3420 cm⁻¹, attributed to residual moisture or hydroxylated metabolites. Strong CH₂ stretching at ~2920/2850 cm⁻¹ reflected typical fatty-acid hydrocarbon chains. At 100 Hz, prominent ester C=O (~1742 cm⁻¹) and =C–H (~3010 cm⁻¹) peaks indicated preserved unsaturation consistent with GC–MS MUFA enrichment. Samples at 1–10 kHz showed moderate C=O intensities and increased C–O stretching (1160–1090 cm⁻¹), suggesting subtle glycerol-backbone rearrangements. The 100 kHz spectrum showed reduced =C–H intensity but sharper CH₂ peaks, consistent with a shift toward higher saturation. At 1 MHz, the ester carbonyl band was strongest, and =C–H bands diminished, indicating pronounced saturation and oxidative modification. Changes in the CH₂/CH₃ bending region (1460–1375 cm⁻¹) and fingerprint region (1200–900 cm⁻¹) further supported frequency-dependent variations in lipid packing and ester functionality.
